# Bounded Adaptive Sensitivity Through Bio-Inspired Digital Hormone Regulation for Emotionally Intelligent UAV Traffic Monitoring

**DOI:** 10.3390/biomimetics11070472

**Published:** 2026-07-06

**Authors:** Mohamed Zaidan, Nafaâ Jabeur, Ahmed Nait Sidi Moh, Tufail Ahmed, Ansar-Ul-Haque Yasar

**Affiliations:** 1 Transportation Research Institute (IMOB), Hasselt University, 3500 Hasselt, Belgium; mohamed.zidan@uhasselt.be (M.Z.); ansar.yasar@uhasselt.be (A.-U.-H.Y.); 2 Department of Computer Science, German University of Technology in Oman (GUtech), Muscat 1816, Oman; 3 Laboratoire d’Analyse des Signaux et des Processus Industriels (LASPI), IUT of Roanne, Jean Monnet University Saint-Étienne, 42334 Roanne Cedex, France; ahmed.nait@univ-st-etienne.fr; 4 College of Engineering, Qatar University, Doha P.O. Box 2713, Qatar

**Keywords:** autonomous UAV, affective computing, bio-inspired control, neuroendocrine-inspired regulation, Digital Hormone Layer, Pull–Push Engine, bounded adaptation, adaptive emotional sensitivity, multi-timescale control, traffic monitoring

## Abstract

Recently introduced affect-driven UAV controllers model behavioral sensitivity (*α*) as a static personality-dependent parameter, overlooking the cumulative influence of prolonged operational context. The Pull–Push Engine (PPE) regulates behavioral responses through bounded temporal integration; however, its effective sensitivity remains fixed during execution, limiting adaptive evolution under cumulative operational exposure. To overcome this limitation, this paper introduces the Digital Hormone Layer (DHL), a bounded neuroendocrine-inspired regulatory mechanism that dynamically modulates the PPE’s effective sensitivity *α*_eff_. In DHL, three scalar hormones inspired by cortisol-, dopamine-, and oxytocin-regulatory motifs accumulate operational context on a medium timescale. In the evaluated scenarios, the behavioral effect is primarily stress-driven, while reward and operator-engagement channels remain architecturally defined but contribute less prominently. Modulation is constrained within a personality envelope by coefficient construction (Personality Preservation Budget (PPB) *ρ* = 0.20). On emergency events, the DHL-augmented controller responds up to 1.91× faster under multi-stressor exposure relative to the activation-selectivity (EI-Low, *α* = 0.495) control (95% bootstrap confidence interval [1.70×, 2.14×]). This indicates the advantage arises from the bounded adaptive DHL trajectory, not from a low steady-state *α*-value. This interpretation is consistent with the implemented per-step hormone-to-sensitivity coupling, observed as an inverse correlation between accumulated stress and effective sensitivity. Mission-final and total in-flight battery consumption are comparable across the single-agent controllers; no battery-efficiency advantage is claimed. A single-equation multi-agent extension shows that increasing the coupling coefficient reduces inter-agent sensitivity distance from 0.00502 (uncoupled, *γ* = 0) to 0.00243 at *γ* = 0.50 (95% CI [0.00225, 0.00261]; a 51.6% reduction; one-way ANOVA F = 82.5, *p* < 0.0001) while both agents remain within the personality envelope, as evidence of bounded inter-agent coupling; system-level multi-agent properties remain to be evaluated.

## 1. Introduction

Autonomous UAVs operating in urban traffic monitoring [1] commonly face conditions that progressively alter the operational context of their missions (e.g., sustained equipment stress, accumulated exposure to high-congestion zones, operator interaction patterns, and multi-hazard sequences). Conventional UAV control strategies span rule-based approaches such as threshold-based congestion monitoring and battery-triggered return-to-base, which provide predictable behavior but lack adaptive response to such cumulative context. Learning-based approaches, including reinforcement learning for UAV flight control [2] and emotion-modulated reinforcement learning [3], extend UAV adaptivity at the cost of extensive training and policies whose internal state is difficult to interpret. As a complementary direction, affect-driven control casts internal regulation as the modulation of bounded behavioral parameters by an interpretable internal state. Within this direction, the Pull–Push Engine (PPE), introduced in [4], emerged as a promising behavioral regulation mechanism that initializes response sensitivity from personality-derived parameters and maintains these sensitivity levels throughout the mission execution. This is principled for personality consistency but does not permit the system to respond to the cumulative operational history that distinguishes a multi-stressor mission from a routine patrol. Consequently, the PPE responds to sustained hazardous exposure at later operational stages using the same sensitivity configuration established at the beginning of the mission, despite the accumulation of prolonged environmental pressure and stress conditions. The central design challenge is therefore to enable bounded behavioral adaptation through deployment-time sensitivity regulation. At the same time, the adaptation must remain, by construction, confined within a predefined behavioral envelope established at mission initialization. Addressing this challenge requires a multi-timescale regulatory organization in which slower adaptive processes influence the responsiveness of faster cognitive and behavioral mechanisms. Such an organization reflects a common principle in biological regulation, where endocrine modulation gradually shapes the sensitivity of faster cognitive and behavioral responses.

Motivated by this principle, we introduce in this paper the Digital Hormone Layer (DHL), a bounded parameter-level regulator that adapts effective emotional sensitivity during mission execution without modifying the established PPE decision structure. Three scalar hormones, drawing on neuroendocrine regulatory principles [5] (stress, reward, and operator-engagement channels), accumulate operational context on a timescale intermediate between per-step Pleasure–Arousal–Dominance (PAD) [6] updates and mission-level personality initialization. Each hormone accumulates under triggering events and decays more slowly between them, encoding a persistent operational context rather than an instantaneous state. Furthermore, the proposed solution introduces a Personality Preservation Budget (PPB) that bounds the sensitivity deviation from the personality baseline, thereby preserving the behavioral identity defined at mission start while allowing context-driven adaptation.

The central empirical finding is that, under sustained multi-stressor exposure, the DHL-augmented controller responds to emergency events markedly faster than a post hoc activation-selectivity control designed to rule out steady-state-*α* explanations. This control does not reproduce the response-time advantage of the adaptive controller, while remaining not significantly different from the standard fixed-*α* baseline. The per-step relationship between accumulated stress and effective sensitivity is observed as a consistent inverse correlation, in line with the implemented hormone-to-sensitivity coupling (full statistical apparatus in Section 5).

The contributions of this work are twofold: (i) bounded sensitivity regulation as a bio-inspired engineering primitive. A parameter-level bounded regulator using neuroendocrine-inspired accumulation-decay dynamics, with coefficient construction guaranteeing that adaptation remains within a predefined personality envelope (the Bounded Effective Sensitivity Property, BESP). (ii) Activation selectivity as a system-level property. This selectivity operates at the level of individual events: emergency events are accelerated while routine events are preserved, and the advantage is present in both single-incident (Sc2) and multi-stressor (Sc3) conditions. The bounded-adaptation property additionally holds under inter-agent coupling in a single-equation multi-agent extension. Together, these contributions deliver a bio-inspired three-timescale affect-driven controller—personality (static), emotion (per-step), hormones (slow context regulation)—within an explicit personality-preservation envelope.

In the remainder of the paper, Section 2 reviews the PPE foundation and bio-inspired control antecedents. Section 3 specifies the DHL architecture and proves BESP. Section 4 details the simulation methodology, scenarios, and the EI-Low-UAV (Emotionally Intelligent UAV with low fixed α) control design. Section 5 and Section 6 report and interpret the empirical results. Section 7 discusses limitations, and Section 8 concludes.

## 2. Related Work

UAV systems have become central to intelligent transportation, supporting urban traffic monitoring and analysis [1], object detection [7], and multi-UAV coordination in natural-disaster and emergency settings [8]. Early multi-agent patrolling research formalized the coverage problem and proposed fixed-path and territory-allocation strategies [9,10]; these assume a known environment with a largely static allocation and are therefore not designed for demand-driven monitoring whose priorities shift over time. A representative smart traffic-monitoring approach [11] improves coverage efficiency but treats the UAV as a utility-maximizing agent with no internal state, leaving no mechanism to weight competing demands by accumulated operational history. At the airspace-management layer, bio-inspired methods have also been proposed for structural design: a recent Slime-Mold-Algorithm approach generates adaptive cyclic UAS corridors through stigmergic swarm agents that are explicitly decoupled from any individual aircraft [12]. Such work adapts the airspace; the present work instead regulates the internal behavioral sensitivity of a single monitoring agent, an orthogonal and complementary problem.

PAD-dimensional affect models [6] have been applied to virtual agents with primary and secondary emotions [13], to computational OCC-PAD-OCEAN affect modeling [14], and to emotion recognition for human–robot interaction [15], with affective HRI surveyed extensively [16]. Across these settings, emotional state is typically treated as an output to be communicated rather than a regulatory signal that shapes internal decision-making. This distinction is central: expressing affect and using affect to bound behavior are different functions, and only the latter is relevant to operational regulation under sustained stress.

The pull/push principle of affect-driven behavioral regulation for traffic-monitoring UAVs was first introduced in our earlier work [17] and subsequently formalized as the Pull–Push Engine (PPE) [4], which balances a personality-derived baseline against environmental appraisal in a single bounded update equation. The PPE operates as a fast-timescale regulator, responding to moment-to-moment appraisal. However, it does not model the slower-timescale modulation of behavioral sensitivity that characterizes biological regulation under accumulated stress, leaving an explicit opening for a complementary layer at that timescale. The present Digital Hormone Layer (DHL) extends [4] at this slow timescale and modifies none of its fast-timescale machinery; the two are layered, not equivalent.

Beyond affective architectures, a broad adaptive-control literature addresses online behavioral adjustment, but along axes distinct from interpretable affective regulation. Adaptive gain scheduling interpolates or switches pre-tuned controller gains across an operating envelope [18,19]; its gain-selection rule carries no internal state representing operational history and selects gains rather than regulating an interpretable sensitivity. Model-reference and L1 adaptive state-feedback control drive tracking error to zero against a reference model under uncertainty [20,21], an objective oriented toward reference-tracking and disturbance rejection rather than toward preserving a behavioral baseline, with adapted parameters that are not designed to be human-interpretable. Meta-control arbitrates between controllers or strategies via cost–benefit signals [22], determining which policy to deploy rather than adjusting policy sensitivity within fixed bounds. Neuromodulated and emotion-modulated reinforcement learning use neuromodulator- or affect-inspired signals to tune learning hyperparameters [3,23] but yield a learned, opaque policy that requires retraining to adapt and offer no deployment-time boundedness guarantee. Recent data-driven UAV control formulations [2] further demonstrate the effectiveness of adaptive learning-based approaches, occupying the data-efficient end of this spectrum with reduced transparency compared with explicitly structured mechanisms. More broadly, the integration of data-driven adaptation with structured modeling principles, namely data-physics fusion [24] and reliability-informed frameworks [25], reflects a field-wide shift away from opaque adjustment toward transparent, constraint-aware adaptation, a direction with which the DHL conceptually aligns; these works are cited to broaden the methodological context rather than as direct methodological antecedents of the DHL. Importantly, these adaptive-control families are designed primarily for performance optimization, tracking, or policy selection, rather than for preserving a stable behavioral identity under adaptation. Within the literature reviewed, none of these families explicitly constrains adaptation within a personality-preservation envelope.

The DHL’s most direct antecedents are bio-inspired neuroendocrine controllers operating on timescales longer than moment-to-moment neural dynamics [5,26]. Cortisol-mediated reactivity suppression under sustained stressor exposure [27] and dopamine-mediated reward sensitization across multiple time courses [28] are canonical slow-timescale neuromodulatory mechanisms, and engineering adaptations have appeared in hormone-based controllers for evolution-minimal robots [29], artificial-endocrine power management in robotic systems [30], hedonic and homeostatic pleasure models for action selection [31], biologically inspired adaptive robot behavior [32], and PAD-grounded affect-driven HRI [33]. These works establish hormone-modulated regulation as viable, but each addresses an isolated element, such as action selection, power management, or expressive behavior, without an explicit personality-preservation constraint or a formalized affective-sensitivity parameter bound to an established emotional architecture.

Existing approaches thus each occupy part of the design space but leave one region unfilled. Adaptive-control methods adapt gains, policies, or learned weights, but not an interpretable affective parameter; affective models express state without bounding behavior; the PPE regulates at the fast timescale only; and prior bio-inspired controllers modulate behavior without an explicit personality envelope. The DHL brings these elements together in a single architecture that prior work has addressed separately: a bounded, interpretable, personality-preserving adaptive sensitivity regulator, operating at the slow neuroendocrine timescale on an established emotional architecture, with interpretable coefficients (k_s_, k_r_, k_c_, ρ). To our knowledge, no prior approach, whether affective, adaptive-control, or bio-inspired, simultaneously occupies the bounded, interpretable, slow-timescale, and personality-preserving region of this design space, which is precisely the region the DHL is constructed to fill. Table 1 summarizes this positioning across the four design-space axes.

## 3. System Architecture

**Figure 1:**
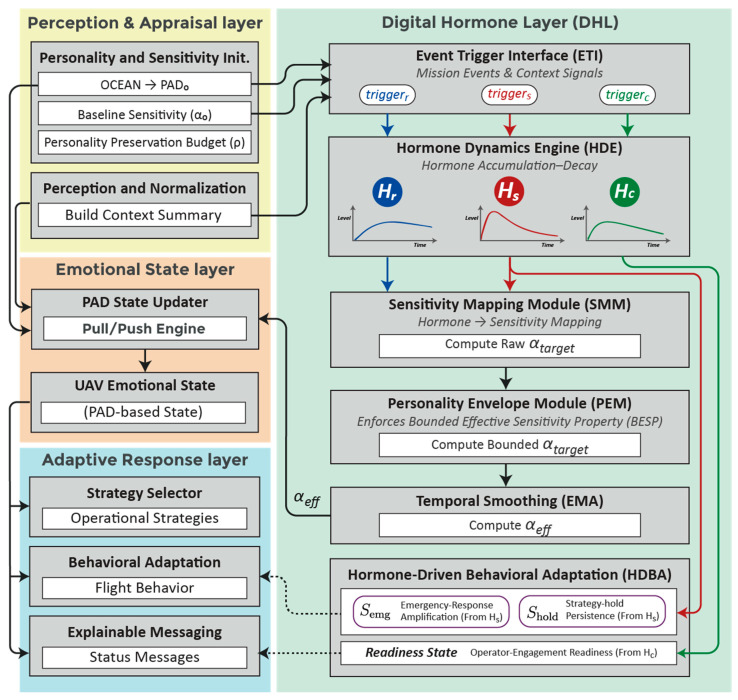
The bounded Digital Hormone Layer (DHL) architecture. **Left**: the prior three-layer host controller [4] (Perception and Appraisal, Emotional State, and Adaptive Response layers). **Right**: the DHL, whose six modules comprise a five-stage sensitivity pipeline—Event Trigger Interface (ETI), Hormone Dynamics Engine (HDE), Sensitivity Mapping Module (SMM), Personality Envelope Module (PEM), and Temporal Smoothing (EMA)—mapping mission events to a personality-bounded effective sensitivity α_eff_ under the Bounded Effective Sensitivity Property (BESP), while preserving the baseline personality configuration. The sixth module, Hormone-Driven Behavioral Adaptation (HDBA), modulates host behavior through bounded channels: emergency-response amplification (S_emg_) and strategy-hold persistence (S_hold_) from the stress hormone H_s_, and operator-engagement readiness modulation from the operator-engagement hormone H_c_. The three hormones are inspired by cortisol-, dopamine-, and oxytocin-regulatory motifs and are implemented as bounded scalar signals rather than biological models. Solid arrows denote the effective-sensitivity regulation path to the PPE; dashed arrows denote the HDBA behavioral-modulation interface.

The proposed system augments a previously published emotionally intelligent UAV control model with a new regulatory layer. Figure 1 shows the complete architecture in two parts. The left part is the prior three-layer model introduced in [4], reproduced here for context and not claimed as a contribution of the present work. The right part is the Digital Hormone Layer (DHL), the contribution of this work, which adapts the controller’s effective sensitivity during a mission while preserving the prior decision structure. The two parts and their interface are described in Section 3.1 and Section 3.2, and each DHL module is then described in turn in Section 3.2.1, Section 3.2.2, Section 3.2.3, Section 3.2.4, Section 3.2.5 and Section 3.2.6.

### 3.1. Prior Architecture Summary

The host controller is the three-layer emotionally intelligent UAV model of [4], shown on the left of Figure 1. It comprises three layers. The Perception and Appraisal Layer appraises mission events through the OCC event model and initializes the Pleasure–Arousal–Dominance (PAD) state from the personality profile: the baseline PAD state PAD_0_ is set from Big Five (OCEAN) parameters [34], and the baseline emotional sensitivity *α*_0_ is the personality baseline used by the controller. All experiments use the Calm personality profile, for which *α*_0_ = 0.57 [4]; this is the fixed-sensitivity baseline of the EI-UAV controller and the central value the DHL modulates. The Emotional State Layer is governed by the Pull–Push Engine (PPE), a three-state regulator (REGULATING, OVERRIDE, RECOVERING) that maintains the PAD trajectory through the bounded update:

(1) *PAD*(*t* + 1) = *PAD*(*t*) + *α* · Δ*ctx* + (1 − *α*) · *β*_0_ · (*PAD*_0_ − *PAD*(*t*))

where Δctx is the environmental appraisal delta from OCC event processing [4,35], PAD_0_ is the personality-derived baseline, β_0_ is the baseline attraction coefficient, and α is the emotional sensitivity. The Adaptive Response Layer maps the current mood to one of eight patrol strategies (S0 to S7) and produces the corresponding flight behavior and status messaging. Full strategy definitions, the mood-to-strategy mapping, and the PPE state-transition logic are specified in [4]; the present work modifies none of these. In prior work, α was fixed at α_0_ throughout a mission; the present work introduces a dynamic effective sensitivity α_eff_ supplied by the DHL, leaving all other PPE parameters unchanged.

### 3.2. Digital Hormone Layer: Overview

The Digital Hormone Layer (DHL), shown on the right of Figure 1, is a parameter-level regulator that adapts the PPE’s effective sensitivity during mission execution without altering the host decision structure. It maintains three scalar hormone states that accumulate operational context on a timescale intermediate between per-step PAD updates and mission-level personality initialization, and it maps these states to a bounded effective sensitivity that replaces the fixed α in Equation (1). The design boundary is deliberately narrow: three scalar state variables are added; the effective sensitivity is the primary controlled quantity and is bounded within a personality envelope of half-width ρ = 0.20; and a small set of auxiliary stress-driven modulations adjust pre-existing controller variables rather than introducing new decision pathways. The PAD update structure, strategy logic, PPE control flow, β_0_, and PAD_0_ all remain identical to [4].

The DHL is organized as a pipeline of six modules, mirrored in Figure 1: an Event Trigger Interface (ETI) that converts mission events and context into hormone triggers; a Hormone Dynamics Engine (HDE) that maintains the three hormone states through accumulation and decay; a Sensitivity Mapping Module (SMM) that forms the raw sensitivity target from the hormone states; a Personality Envelope Module (PEM) that enforces the Bounded Effective Sensitivity Property (BESP); a Temporal Smoothing (EMA) module that produces the effective sensitivity supplied to the PPE; and a Hormone-Driven Behavioral Adaptation (HDBA) module that applies bounded stress-driven and engagement-driven modulations to existing behavioral parameters. The DHL connects to the host model at two interfaces: the effective sensitivity α_eff_ replaces α in the PPE (Equation (1)), and the HDBA channels act on the Adaptive Response Layer’s behavioral output and messaging. The six modules are described in turn below. The DHL adds a small set of scalar state variables and does not alter the host decision structure (per agent: H_s_, H_r_, H_c_, α_eff_), leaving the host PPE decision structure unchanged. Algorithm 1 traces the full per-step procedure across these six modules.
**Algorithm 1.** Mission-step execution flow of the DHL within the host PPE controller.Input: hormone states (Hs, Hr, Hc); current effective sensitivity αeff; personality baseline α_0_; active triggers {T1…T4}; current event; step index tOutput: updated sensitivity αeff′; emergency factor S_emg; updated hormones (Hs′, Hr′, Hc′)Each step traces one mission step through the six DHL modules of Figure 1 (Section 3.2.1, Section 3.2.2, Section 3.2.3, Section 3.2.4, Section 3.2.5 and Section 3.2.6), ending at the PPE host-controller hand-off.1. [ETI → HDE] Read the active mission triggers at step t and update the three hormones (Equation (2a–c)): Hs rises quickly and decays slowly; Hr and Hc follow their exponential-decay channels. All three are clipped to [0,1] (the BESP precondition).▸ accumulate hormones2. [SMM → PEM] Map Hs and Hr to a target sensitivity (Equation (3)); Hc does not enter the sensitivity path and instead drives the operator-focus channel (Section 3.2.6). The target sensitivity remains inside the personality envelope by construction; the CLAMP acts only as a redundant safety net.▸ bounded target3. [EMA] Every 100 steps, move αeff one first-order step toward the target (Equation (4)); hold it constant between updates. This slow time-scale separates sensitivity drift from per-step dynamics.▸ smoothing4. [HDBA] If the current event is a discrete OCC emergency, set the emergency-response factor from accumulated stress (Equation (5)), S_emg ∈ [1.00, 1.30]; otherwise, S_emg = 1 under routine patrol. (The stress-driven dwell scaling S_dwell and strategy-hold scaling S_hold, and the Hc operator-focus gain γ_op—all Section 3.2.6—are secondary behavioral channels applied in the PPE and are omitted here for clarity.)▸ emergency gain5. [→ PPE] Hand αeff′ to the PPE host-controller update [4] in place of the fixed α and apply S_emg to the emergency-detection gain. The PPE decision structure is otherwise unchanged. The resulting αeff′ becomes the sensitivity parameter for the next PPE update cycle.▸ PPE hand-offreturn (αeff′, S_emg, Hs′, Hr′, Hc′)Note: Hormones are bounded to [0,1], and αeff remains inside the personality envelope by construction—guaranteed by the coefficient construction ks = kr = ρ·α_0_ (Section 3.2.4), not by the CLAMP. Parameter values are listed in Table 2.

#### 3.2.1 Event Trigger Interface (ETI)

**Function.** The ETI converts mission events and context signals into the per-channel trigger contributions that drive hormone accumulation. The ETI produces three trigger signals, one per hormone channel: trigger_s (stress), trigger_r (reward), and trigger_c (operator-engagement).

The stress channel is driven by four discrete triggers (T1 to T4) reflecting distinct operational stressors. T1 fires on sustained high-congestion exposure (at least three consecutive steps in High or Severe congestion, with a 20-step refractory period) and contributes +0.8 per fire. T2 fires when at least two emergency events accumulate within a 500-step rolling window (edge-triggered) and contributes +1.0. T3 fires when battery falls below 40% (one-shot per recharge cycle, re-armed at battery at least 60%) and contributes +0.5. T4 fires on prolonged operator-command intervention (at least three commands within a 300-step window, edge-triggered) and contributes +0.6. The reward trigger fires after successful zone-coverage events, and the operator-engagement channel integrates negative-mood (distress) episodes, rising under sustained distress and decaying as mood recovers, with operator intervention accelerating clearance. Under the Bologna network’s baseline congestion profile, the congestion trigger T1 dominates accumulation, so the stress state is driven toward its operating range on incident-free missions as well.

**Data flow.** Mission events and context signals enter the ETI; the resulting trigger_s, trigger_r, and trigger_c are passed to the Hormone Dynamics Engine (Section 3.2.2).

#### 3.2.2. Hormone Dynamics Engine (HDE)

**Function.** The HDE maintains the three scalar hormone states through accumulation under triggers and decay between them, encoding a persistent operational context rather than an instantaneous state. The three channels are slow accumulation-decay regulators with distinct activation contexts; the hormone names are used as channel labels only.

The stress hormone follows linear first-order accumulation and decay:

(2a) *H_s_*(*t* + 1) = *H_s_*(*t*) + *δ_s_* · *trigger_s_*(*t*) − *λ_s_* · *H_s_*(*t*)

The reward and operator-engagement hormones follow accumulation with exponential decay:

(2b) *H_r_*(*t* + 1) = (*H_r_*(*t*) + *δ_r_* · *trigger_r_*(*t*)) · *exp*(−1/*τ_r_*)

(2c) *H_c*(*t* + 1) = (*H_c*(*t*) + *δ_c* · *trigger_c*(*t*)) · *exp*(−1/*τ_c*)

with stress parameters δ_s_ = 0.10 and λ_s_ = 0.005, reward parameters δ_r_ = 0.10 and τ_r_ = 100, and operator-engagement parameters δ_c_ = 0.03 and τ_c_ = 400 (Table 2). After each update, all hormone states are clipped to [0,1], enforcing the domain precondition of the BESP (Section 3.2.4). The asymmetry λ_s_ ≪ δ_s_ is deliberate: fast accumulation under active hazards with slow decay encodes persistent context memory, so a history of sustained stress continues to modulate sensitivity even during brief recovery intervals.

The evaluated scenarios primarily activate the stress pathway; the reward and operator-engagement channels are retained as bounded architectural channels and are discussed where relevant (Section 5.5 and Section 7). The three hormones are introduced as an architectural design primitive, not as three empirically co-validated channels: the BESP (Section 3.2.4) bounds the joint contribution of H_s_ and H_r_ for any admissible hormone state, while empirical evaluation here concentrates on the stress pathway because the evaluated scenarios engage it as the dominant active channel while leaving reward and operator-engagement subdominant (Section 5.5).

**Data flow.** The HDE receives triggers from the ETI and outputs the current hormone states H_s, H_r, and H_c. H_s and H_r are passed to the Sensitivity Mapping Module (Section 3.2.3); H_s and H_c are also passed to the HDBA module (Section 3.2.6).

#### 3.2.3. Sensitivity Mapping Module (SMM)

**Function.** Forms the raw sensitivity target from the hormone states, combining the baseline sensitivity with stress-driven reduction and reward-driven elevation.

(3) *α_target* = *clip*(*α*_0_ − *k_s_* · *H_s_* + *k_r_* · *H_r_*, [*α*_0_(1 − *ρ*), *α*_0_(1 + *ρ*)])

with k_s_ = k_r_ = ρ · α_0_ = 0.114 (Table 2). The stress hormone reduces the sensitivity target and the reward hormone raises it; the coefficient construction k_s_ = k_r_ = ρ · α_0_ is what guarantees the bound enforced by the next module. The clip shown in Equation (3) is an adversarial-conditions safety net rather than the primary bounding mechanism, which is the coefficient construction itself (Section 3.2.4). In implementation, the sensitivity target responds to each hormone’s deviation from a slowly adapting internal baseline, so sustained hormone levels are absorbed into the baseline, whereas relative increases or decreases transiently shift αeff within the bounded envelope. The operator-engagement hormone H_c_ does not enter Equation (3); it acts through the HDBA module (Section 3.2.6).

**Data flow.** The SMM receives H_s and H_r from the HDE and outputs the raw target alpha_target to the Personality Envelope Module (Section 3.2.4).

#### 3.2.4. Personality Envelope Module (PEM) and the Bounded Effective Sensitivity Property

**Function.** The PEM enforces the Bounded Effective Sensitivity Property (BESP): it keeps the sensitivity target and the smoothed effective sensitivity derived from it within the personality envelope for all admissible hormone states.

The personality envelope is [α_0_ (1 − ρ), α_0_ (1 + ρ)]. Substituting α_0_ = 0.57 and ρ = 0.20 gives the hard bounds [0.456, 0.684].

**BESP.** Given H_s_, H_r_ in [0,1] and k_s_ = k_r_ = ρ · α_0_, both the unclipped target α_target = α_0_ − k_s_ · H_s_ + k_r_ · H_r_ and the smoothed α_eff_ of Equation (4) are contained in the envelope for all admissible hormone states and all t ≥ 0. **Proof.** The target is linear in H_s_ and H_r_. Substituting the coefficient construction, the minimum occurs at (H_s_, H_r_) = (1, 0), yielding α_0_ (1 − ρ), and the maximum at (0, 1), yielding α_0_ (1 + ρ). By linearity, all (H_s_, H_r_) in [0,1]^2^ give a target inside the envelope. The clip enforces the same bounds if hormone values ever exceed their nominal range. For the smoothed α_eff_: since each target lies in the envelope and α_eff_ (t + 1) is a convex combination of α_eff_ (t) and the current target, with α_eff_ (0) = α_0_ inside the envelope, induction on t keeps α_eff_ inside the envelope for all t. This guarantees bounded effective sensitivity by construction. It does not by itself constitute a full closed-loop stability proof for UAV motion or mission-level safety.

**Data flow.** The PEM receives alpha_target from the SMM and outputs the bounded target to the Temporal Smoothing module (Section 3.2.5). The operator-engagement envelope is bounded separately by H_c in [0,1] and the fixed coefficient k_c (Section 3.2.6).

#### 3.2.5. Temporal Smoothing (EMA)

**Function.** The EMA produces the effective sensitivity that drives the PPE by slow first-order smoothing of the bounded target, enforcing time-scale separation between the hormonal layer and the per-step PAD dynamics.

(4) *α_eff*(*t* + 1) = (1 − *λ*) · *α_eff*(*t*) + *λ* · *α_target*(*t*)

with smoothing rate λ = 0.10, applied every 100 simulation steps; between updates, α_eff_ is held constant. The 100-step update interval, combined with the slow smoothing, enforces time-scale separation between the hormonal layer and the per-step PAD dynamics and is the source of the cadence-induced lag between the formula target and the stored effective sensitivity that drives the controller, characterized quantitatively in Section 5.4. The resulting α_eff_ replaces α in the PPE update (Equation (1)); the existing mood-to-strategy mapping is applied unchanged.

**Data flow.** The EMA receives the bounded target from the PEM and outputs alpha_eff to the PPE in the Emotional State Layer (the right-to-left interface in Figure 1).

#### 3.2.6. Hormone-Driven Behavioral Adaptation (HDBA)

**Function.** The HDBA applies bounded stress-driven and engagement-driven modulations to pre existing behavioral parameters of the Adaptive Response Layer. Each modulation is a multiplicative scaling on a quantity already present in the host controller; no new decision pathway is introduced. The stress-driven channels are bounded by construction since the stress hormone lies in [0,1]; the engagement channel is bounded by the operator-engagement hormone in [0,1] and a fixed coefficient.

**Emergency-response amplification (S_emg_, from H_s_).** When a discrete OCC emergency event (for example, an Accident) is registered, the controller’s detection gain is scaled by

(5) *S_emg* = 1 + *k_emg* · *H_s_*

with k_emg_ = 0.30 (Table 2), giving S_emg_ in [1.00, 1.30]. The factor is applied only when an emergency event fires and is inert under routine patrol (S_emg_ = 1 otherwise).

**Congestion-aware dwell scaling (S_dwell_, from H_s_).** The per-zone dwell threshold is scaled differentially by congestion class:

(6a) *S_dwell* = 1 + *k_high* · *H_s_*          (High or Severe)

(6b) *S_dwell* = *max*(0.40, 1 − *k_low* · *H_s_*)          (*Low or Moderate*)

with k_high_ = 0.80, k_low_ = 0.60, and a hard floor of 0.40. S_dwell scales the per-zone dwell threshold upward in High and Severe congestion and downward in Low and Moderate congestion, both bounded; its empirical contribution is characterized in the leave-one-out analysis of Section 5.

**Strategy-hold persistence (S_hold_, from H_s_).** The minimum hold time before a strategy switch is scaled by

(7) *S_hold* = 1 + *k_T* · *H_s_*

with k_T_ = 1.0, applied to the base strategy-hold duration, producing greater strategy inertia under stress while leaving routine behavior unchanged (S_hold_ = 1 at H_s_ = 0). Safety overrides, such as return-to-base locks, bypass this guard. This channel adjusts strategy-switch timing; its individual contribution to emergency response time is characterized in the leave-one-out analysis of Section 5.

**Operator-engagement readiness state (from H_c_).** The operator-engagement hormone H_c_ sets an operator-engagement readiness state that acts on the operator-focus channel rather than on α_eff_. When an operator-focused event is active, H_c_ amplifies the operator-channel influence on the PPE through a multiplicative gain γ_op_ = 1 + k_c_ · H_c_ with k_c_ = 0.30, applied to the operator-event contributions defined in [4]. This hormone does not enter the effective-sensitivity equation; it acts only on the operator-focus channel and the associated status messaging, leaving α_eff_ for general environmental responsiveness.

**Data flow.** The HDBA receives H_s and H_c from the HDE; its stress-driven channels (S_emg, S_dwell, S_hold) act on Flight Behavior in the Adaptive Response Layer, and its operator-engagement readiness state acts on the Explainable Messaging output. The reward and stress hormones otherwise influence behavior entirely through alpha_eff (Equations (3) and (4)).

### 3.3. Multi-Agent Stress Coupling

**Function.** It extends the single-agent DHL to two agents through a single coupling equation, introducing only one additional scalar parameter.

For two agents i and j sharing an operational region, each agent’s stress hormone is replaced at update time by a coupled variant:

(8) *H*′*_s,i* = (1 − *γ*) · *H_s,i_* + *γ* · *H_s*,*j_*

with γ = 0.50 in the present evaluation (balanced symmetric coupling). The coupled value is used in place of H_s,i_ in Equation (3); all other DHL components are unchanged. Each agent’s envelope (Equation (3) bounds) remains binding independently, so coupling does not push either agent outside the personality envelope. The MHR-UAV (Multi-agent Hormone-Regulated UAV) controller applies this coupling to the DHL sensitivity-regulation core across both agents. The robustness of the Bounded Effective Sensitivity Property under varied coupling strength is evaluated in Section 6.4.

## 4. Materials and Methods

### 4.1. Simulation Environment

Experiments use the SUMO (Eclipse SUMO v1.22.0; German Aerospace Center (DLR), Berlin, Germany) Bologna simulator-of-record [36,37] introduced in [4], with eight monitoring zones (A-H), 6000-step missions, deterministic seeding, and a return-to-base recharge cycle triggered at 20% battery [4]—a conservative safety floor sized to leave sufficient charge for the UAV to return to the recharge dock from any zone in the Bologna Acosta arterial network under nominal energy conditions, applied uniformly across all controllers as a hard safety override. Full network topology, zone coordinates, and scenario event timing are reported in Appendix A. Per-controller recharge counts are reported in Section 5.3. The present study extends [4] to a fully matched six-controller comparison: the same 24 random seeds drive all six controllers in all three scenarios, producing a 24 × 6 × 3 paired design with identical event-injection timelines (e.g., Sc3 Run 1 Accident at step 583 across all controllers). This isolates controller effects from seed-level stochastic variation. The full corpus comprises 432 runs (six controllers in all three scenarios at 24 seeds, including the EI-Low-UAV activation-selectivity control; the rationale and post hoc status of the EI-Low control are discussed in Section 4.2 and Section 5.6). Reported net-consumption values measure mission-final battery consumption rather than total in-flight energy expenditure. The full energy decomposition is reported in Table A3 (Appendix B).

Simulator parameters. Each simulation step represents one second of simulated time (SUMO default). Each mission begins at 100% battery; the recharge cycle restores the battery to 100% on return to base. The base station is co-located with zone A. Recharge cycles take up to 500 simulation steps to restore from 20% to 100% battery (linear restoration at 0.2% per step); cycles terminate early when the battery reaches 100%. Zone radius is 80 m. Congestion classes are defined by vehicle-count thresholds in the 80 m monitoring radius, calibrated for the Bologna network: Severe (≥8 vehicles), High (4–7), Moderate (2–3), Low (0–1). The Bologna Acosta arterial network defines the underlying road topology, with eight monitoring zones (A–H) covering the principal arterial intersections; full network specifications are reported in [4].

### 4.2. Controllers

The controller configurations are summarized in Table 3. ABL-UAV serves as a baseline-restoration ablation condition: the PPE update equation is applied with α fixed at 1.0, removing the personality-restoration term entirely and isolating the contribution of the PPE’s baseline-attraction mechanism. EI-Low-UAV serves as an activation-selectivity control: it is identical to EI-UAV in every respect except that α is fixed at 0.495—below the EIH-UAV Sc3 operating band [0.534, 0.572], i.e., a static value more favorable than EIH-UAV’s own to the lower-α-produces-faster-response hypothesis. EI-Low-UAV isolates the effect of a fixed low α_eff_ without dynamic modulation; if the advantage were a steady-state-α effect, this control should reproduce or exceed it. RBplus-UAV represents an event-aware rule-based baseline designed to approximate practical reactive deployment behavior: a rule-based controller informed by event types that deploys S6 immediately upon accident detection.

Post hoc status of the EI-Low control. The EI-Low-UAV was introduced after the main study evaluation to test the steady-state-α explanation against the dynamic-trajectory account. Its fixed α (0.495) was set before the EI-Low simulations were run, but after the main study EIH data had been observed. The EIH-vs-EI-Low Sc3 response-time comparison is therefore treated as a targeted follow-up test rather than a formally pre-registered prediction; we report it without family-wise correction and disclose its post hoc origin here, in Section 5.6 and Section 7. The remaining six pairwise contrasts are reported as exploratory.

### 4.3. Evaluation Scenarios

The empirical evaluation uses three scenarios of increasing stressor complexity, summarized in Table 4.

Sc3 events are injected at fixed steps with seed-based jitter: Accident (500), SignalLoss (1500), OperatorFocus (2000), Overheated (2600), BadWeather (3000). Per-event uniform jitter of ±100 steps is applied per seed; the realized Accident injection range across the 24 Sc3 seeds is steps 402 to 589. Event durations range from 150 to 500 steps. Temporal spacing produces overlapping events that prevent UAV recovery between incidents. Baseline congestion exposure from the SUMO Bologna traffic load remains active across all three scenarios, driving H_s_ to its scenario-dependent de-saturated band of approximately 0.20–0.28 (Section 5.4).

Per-event specifications. Accident: 500-step duration, blocks two lanes at Zone F, triggering severe congestion classification at the injection point. SignalLoss: 150-step duration, triggers immediate return-to-base for all controllers and disables the operator-link channel for the duration. OperatorFocus: 200-step duration, focuses on Zone C and activates the operator-engagement hormone H_c_. Overheated: 200-step duration, triggers return-to-base unless the UAV is in a post-recharge hold; contributes to H_s_ accumulation. BadWeather: 200-step duration, triggers immediate return-to-base and affects environmental perception.

### 4.4. Evaluation Metrics

Performance was assessed across five metrics: (i) Emergency Response Time (seconds from event injection to UAV arrival at target zone), i.e., the incident-response latency of the monitoring system, the interval from event onset to on-scene arrival; (ii) Severe-zone dwell percentage (SZD%, defined formally below in Equation (9)); (iii) Battery Consumption (net battery percentage consumed per run, measured as the difference between starting and final battery state); (iv) Strategy Entropy (Shannon entropy of the per-run strategy distribution in bits); (v) α_eff_ Dynamics (directly logged effective-sensitivity trajectory).

(9) *SZD*% = 100 · (*Σ Dwell_Steps* [*Severe*])/(Σ Dwell_Steps [all zones])

where Dwell_Steps [Severe] denotes the realized dwell duration in zones whose instantaneous congestion classification is Severe, summed over the full 6000-step run, and Dwell_Steps [all zones] is the corresponding sum over all eight zones (A–H), including the base-station zone A.

Each scenario uses twenty-four runs per controller, yielding approximately 13,400 paired (H_s_, α_eff_) observations per scenario for the EIH controller (24 runs × 600 sampled steps = 14,400 nominal, less approximately 44 observations per run during the ~440-step return-to-base recharge cycle when EIH telemetry is paused). The per-step Pearson correlation in Section 5.4 therefore draws on a larger nominal sample than the per-run aggregate; within-run autocorrelation reduces the effective sample size, so it is reported descriptively rather than as an independent test. Aggregate response-time hypothesis testing uses non-parametric Mann–Whitney U with rank-biserial effect sizes and bootstrap confidence intervals on ratios. Trade-offs of this sample structure are discussed in Section 7.

Statistical significance was assessed using the Mann–Whitney U test. One-sided testing is appropriate for the directional architectural hypothesis that adaptive controllers should not be slower than fixed-α controllers. Both one-sided and two-sided *p*-values are reported in Section 5.1. The comparisons in Section 5.1 are demarcated to control multiple-testing exposure: the EIH-vs-EI-Low Sc3 response-time test is treated as the primary targeted test of the activation-selectivity hypothesis, while comparisons against RB, RBplus, EI, ABL, and MHR are reported as exploratory descriptive contrasts. Under this demarcation, the headline targeted test stands without family-wise correction. Exploratory *p*-values should be interpreted with caution against an uncorrected family-wise error rate of approximately 0.26 across six pairwise tests. Two-sided tests are used for non-distinguishability and architectural-equality claims (i.e., claims that two controllers do not differ in a measurable direction, supported by a non-significant two-sided test result rather than a formal equivalence-margin test).

The 95% confidence interval on the response-time ratio between controllers is computed by non-parametric stratified bootstrap with 20,000 resamples within scenario, with each group resampled independently to accommodate unequal group sizes where they arise after instant-response exclusion; the single-agent cells are balanced at *n* = 24 per controller in Sc2 and Sc3. The ratio of bootstrap means is used as the test statistic, with rank-biserial r_pb reported as a complementary effect-size measure. Of the twenty-four runs per controller-scenario cell, all twenty-four produced non-instant accident responses. Instant responses (UAV already at the target zone at injection) are excluded from response-time aggregates as they reflect patrol positioning rather than reactive navigation. The exclusion distribution and its mechanistic interpretation are reported in Section 5.1. A sensitivity analysis treating instant responses as 0 s responses, rather than excluding them, is reported in Section 5.6 to confirm robustness of the headline ratio to exclusion-criterion choices.

For the EIH single-agent dynamics analysis, α_eff_ is logged at every simulation step, while H_s_, H_r_, and H_c_ are logged every 10 simulation steps. Pearson correlations between H_s_ and α_eff_ are computed at the common 10-step sampling, pooled across all EIH simulation steps within each scenario (n ≈ 13,400 paired observations per scenario). The α_eff_ and H_s_ coupling traces for the MHR-UAV configuration are written to a separate per-run log at the 100-step α_eff_ update interval (Section 3.2.5), producing the paired observations reported in Section 6.4.

## 5. Results

### 5.1. Activation Selectivity and Emergency Response

A central system property of the DHL is activation selectivity. Hormone accumulation and α_eff_ modulation are present in all scenarios and the behavioral signature of that modulation, measured as response-time separation from fixed-α EI-UAV control, is concentrated on emergency events and is present in both scenarios (Sc2 1.54× faster, Sc3 1.84× faster, both *p* < 0.001; Section 6.3). Per emergency type, the acceleration is severity-ordered—Accident 1.41×, SignalLoss 1.91×, Overheated 2.29×—while routine BadWeather is 1.21× (*p* = 0.27, not significant). Because α_eff_ itself is essentially scenario-invariant (≈0.55 across Sc1, Sc2, Sc3; grand-mean 0.553), the activation selectivity reflects whether the same underlying α_eff_ suppression translates into measurable timing advantage, not whether α_eff_ modulation differs across scenarios. In Sc2, EIH-UAV (28.5 s) responds 1.54× faster than EI-UAV (44.0 s) on emergency events (Mann–Whitney *p* < 0.001), and a comparable separation is present in Sc3 (1.84×, *p* < 0.001). The DHL therefore preserves baseline behavioral characteristics on routine activity while preferentially accelerating emergency events wherever they occur, including under the single-incident conditions of Sc2. This selectivity is a designed property that prevents adaptation from producing aggressive behavioral change when context does not warrant it.

Under sustained multi-stressor conditions (Sc3), DHL adaptation produces measurable separation supported by four converging measures of the same response-time distribution, complemented by the per-step mechanism layer, consistent with the implemented hormone-to-sensitivity coupling. Per-cell n after instant-response exclusion (Sc2/Sc3): RB 24/24, RBplus 24/24, EI 24/24, EI-Low 24/24, ABL 24/24, EIH 24/24, MHR 12/10. Magnitude: the EIH-UAV mean emergency-event response time of 26.8 s is 1.91× faster than the EI-Low-UAV mean of 51.1 s (EI-Low is the post hoc activation-selectivity control with α fixed at 0.495; rationale and post hoc status discussed in Section 4.2 and Section 5.6) and 1.84× faster than the standard EI-UAV mean of 49.2 s.

**Figure 2:**
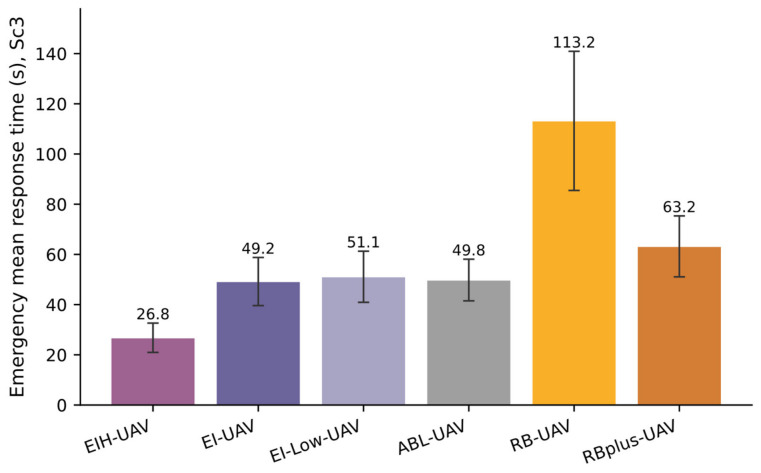
Mean emergency response time per controller (s), with SD error bars, Sc3 (3-type emergency events: Accident, SignalLoss, Overheated; instant responses excluded; run-level, *n* = 24). EIH-UAV 26.8 s versus EI-UAV 49.2 s (1.84× faster); all six single-agent controllers shown.

Effect size: rank-biserial r_pb = 0.965 versus EI-Low (large). Bootstrap 95% CI on the EI-Low/EIH ratio: [1.70×, 2.14×]. Mann–Whitney *p* = 1.0 × 10^−8^ (two-sided) for EIH vs. EI-Low. Section 5.4 per-step layer is consistent with this separation. Table 5 reports response times across all seven controllers; Figure 2 annotates the Sc3 EIH-vs-EI-Low primary targeted test (Section 5.6). The selectivity is event-level: emergency events are accelerated relative to the standard fixed-α (EI) control (Sc3 accident 1.41×, signal-loss 1.91×, Overheated 2.29×, all *p* < 0.01), while routine events are not (BadWeather 1.21×, not significant at *p* = 0.27). The response-time advantage is present in both scenarios against the same EI baseline (Sc2 1.54×, Sc3 1.84×, both *p* < 0.001), with no significant difference in the ratio between them.

Both fixed-α controllers—EI at α = 0.57 and EI-Low at α = 0.495—are not significantly different from each other in Sc3 response time (*p* = 0.47 two-sided, r_pb = 0.12).

EIH-UAV is also faster than RBplus-UAV (63.2 s, 2.36×, *p* < 0.001) and RB-UAV (113.2 s, 4.23×, *p* < 0.001). Both fixed-α emotional controllers (EI and EI-Low) are themselves significantly faster than RBplus-UAV (1.28× and 1.24×, respectively, *p* < 0.01). These results suggest a two-level pattern within the evaluated controller set: PAD-based emotional controllers outperform the tested rule-based baselines, while dynamic α modulation further improves response time over the evaluated fixed-α emotional controllers.

MHR-UAV maintains emergency response substantially faster than the rule-based baselines (49.8 s vs. RB 113.2 s, *p* < 0.001). Because the multi-agent and single-agent results are drawn from separate campaigns, no direct comparison between them is asserted; the bounded-coupling interpretation is given in Section 6.4.

### 5.2. Severe Zone Coverage

Severe-zone dwell percentages across controllers and scenarios are summarized in Table 6.

EIH-UAV achieves 45.7% SZD (Equation (9)) in Sc3, at parity with EI-UAV (41.6%; Mann–Whitney *p* = 0.06, rank-biserial r = 0.31, 95% CI [−0.01, 0.61]) and exceeding the competing rule-based baselines by 2.4–4.2× (RB: 10.8%, 4.2×; RBplus: 18.7%, 2.4×). SZD parity between EIH and fixed-α emotional control is the predicted outcome under bounded adaptation: the personality envelope (ρ = 0.20) limits the maximum sensitivity excursion from α_0_, and the SZD% metric reflects sustained spatial occupancy that does not require unbounded reactivity to achieve comparable severe-zone occupancy. The Sc3 advantage of the DHL is therefore concentrated in metrics that reward dynamic adaptation (response time; Section 5.1 and Section 5.3) rather than in steady-state coverage, by architectural design. The EI-Low-UAV control (42.4% Sc3 SZD) does not differ significantly from EI-UAV (41.6%; Mann–Whitney *p* = 0.78, two-sided; rank-biserial r = 0.05, 95% CI [−0.30, 0.38]), confirming that Severe-zone dwell in Sc3 is similarly insensitive to whether α is fixed at 0.57 or at 0.495. Coverage parity holds across both fixed-α configurations and the adaptive EIH controller.

A noteworthy observation is the EIH-vs.-ABL comparison: ABL-UAV (45.8%) reaches Severe-zone dwell not significantly different from EIH-UAV (*p* = 0.99) despite removing the personality-restoration term entirely (α = 1.0), the DHL therefore matches unbounded reactivity on Severe-zone occupancy while keeping α_eff_ within ±20% of α_0_ (ρ = 0.20).

MHR-UAV Sc3 Severe-zone dwell (21.5%) is lower than the single-agent controllers, consistent with zone-splitting between the two coupled agents reducing per-agent persistence at any single Severe zone. With no incidents to concentrate dwell, the agents distribute across zones, and neither commits to the single most-Severe area as persistently as a single agent does. Under Sc3 stressor exposure, MHR per-agent Severe-zone dwell (21.5%) exceeds the rule-based baselines (RB 10.8%). Because the multi-agent and single-agent results are drawn from separate campaigns, no direct comparison is asserted (Section 6.4). Additional analysis of worst-case emergency response is reported in Appendix C.

### 5.3. Mission-Final Battery Consumption

Mission-final battery consumption remains within a narrow range across the single-agent controllers (Table 7). In Sc1 and Sc2, all controllers consume similar baseline energy (≈56–59%). Under multi-stressor load (Sc3), consumption rises across the board into a 71.0–75.0% band: the rule-based controllers consume 71.0–71.1%, while EI-UAV (74.1%), EI-Low-UAV (74.1%), ABL-UAV (74.2%), and EIH-UAV (75.0%, ±1.5 SD) cluster at the upper end. Because the per-step drain is identical by construction, total in-flight energy remains similar across single-agent controllers (Table A3), and no battery-efficiency advantage is claimed; the remaining differences in net mission-final consumption reflect recharge-cycle timing rather than efficiency differences. This interpretation is consistent with the observed recharge frequencies (approximately one recharge per run for EI, EI-Low, ABL, and EIH; 2.6–2.8 for the rule-based controllers). MHR-UAV consumes 62.4% at Sc3, a separate-campaign readout reported without between-controller interpretation.

**Table 7:** Mission-final battery consumption (net of recharge cycles). Net percentage consumed per run, mean ± SD.

Scenario	RB-UAV	RBplus-UAV	EI-UAV	EI-Low-UAV	ABL-UAV	EIH-UAV	MHR-UAV
Sc1	57.5 ± 0.2	58.4 ± 0.4	58.8 ± 0.1	58.8 ± 0.1	58.5 ± 0.1	58.8 ± 0.1	55.6 ± 0.3
Sc2	57.5 ± 0.2	57.4 ± 0.6	58.6 ± 0.2	58.6 ± 0.2	58.7 ± 0.2	58.8 ± 0.1	55.6 ± 0.2
Sc3	71.0 ± 1.3	71.1 ± 1.1	74.1 ± 1.7	74.1 ± 1.6	74.2 ± 1.2	75.0 ± 1.5	62.4 ± 6.0

### 5.4. Implementation-Consistency of the α_eff_ Dynamics

Table 8 presents directly logged α_eff_ statistics for the EIH-UAV controller, with per-step Pearson correlations between accumulated stress and effective sensitivity computed from ≈ 13,400 paired observations per scenario; because successive steps are strongly autocorrelated (lag-1 r ≈ 0.99), the effective sample is far smaller, so this per-step relationship is reported descriptively, not as an independent test. The per-step range column in Table 8 reports min/max α_eff_ across all timesteps in all twenty-four runs; the upper bound equals α_0_ since each run begins at α_eff_ = α_0_ before H_s_ accumulates.

Two observations characterize the implemented coupling. First, α_eff_ is measurably reduced from α_0_ across all scenarios (−1.8% to −3.5%, grand-mean −2.9%), a reduction that does not scale linearly with injected-stressor count. With mean H_s_ ≈ 0.248 and k_s_ = ρ · α_0_ = 0.114, the nominal prediction α_0_—k_s_·0.248 = 0.542 differs from the observed 6000-step time-averaged value of 0.553 by 0.011, reflecting the early-mission warmup ramp and the EMA update lag (λ = 0.10). Per-scenario H_s_ means lie in a de-saturated band (Sc1 0.195, Sc2 0.279, Sc3 0.270): adding stressors from Sc2 to Sc3 does not raise the mean (both ≈ 0.27), consistent with the H_s_ trigger structure (Section 3.2.1, sustained operational load including baseline congestion rather than emergency incident stress alone).

Second, the Pearson correlation between accumulated H_s_ and effective sensitivity is consistently inverse: r = −0.79 (Sc1), −0.60 (Sc2), −0.45 (Sc3); the coupling weakens as multi-event load fragments the stress trajectory. These per-step correlations are reported as descriptive characterizations of the implemented hormone-to-sensitivity coupling, not as inferential confirmation.

Because α_eff_ is generated deterministically from H_s_ through the target-and-EMA update (Equations (3) and (4)), the contemporaneous correlation is expected by construction and cannot serve as independent evidence. To characterize the implemented relationship, we ran three run-level time-series analyses on the twenty-four Sc3 trajectories. (i) The H_s_–α_eff_ cross-correlation is maximized at a positive lag of approximately 440 simulation steps (H_s_ leading α_eff_, peak r = −0.76) and decays toward zero or reverses at negative lags, so α_eff_ tracks accumulated past stress with the smoothing memory imposed by the EMA rather than anticipating it. (ii) After removing a quadratic time trend from both series, the partial correlation remains inverse (−0.31, −0.10, and −0.28 for Sc1, Sc2, and Sc3), so the coupling is not an artifact of co-drift with mission time. (iii) Granger tests on first-differenced series are significant for H_s_ → α_eff_ in 15 of 24 Sc3 runs (23 of 24 in Sc1) but for the reverse α_eff_ → H_s_ direction in only 1 of 24, a directional asymmetry consistent with the one-way construction. These analyses confirm that the controller realizes the specified directional, lagged, low-pass H_s_-to-α_eff_ transfer; they are implementation-consistency evidence, verifying that the implementation follows Equations (3) and (4), and not independent mechanistic validation. The operational value of the stress-driven sensitivity channel is established separately by the channel-ablation analysis and the fixed-α frontier, not by this correlation.

**Figure 3:**
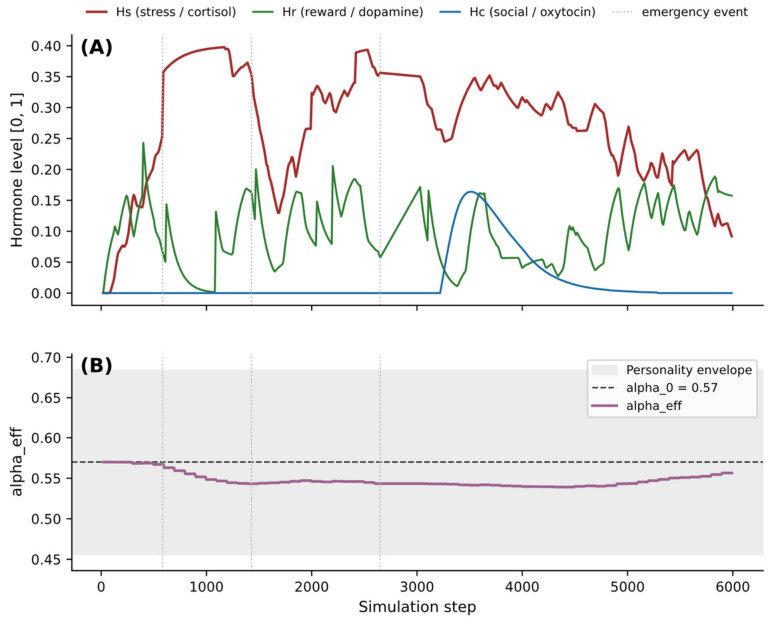
Hormone machinery, representative Sc3 EIH-UAV run. (**A**) H_s_, H_r_, H_c_ trajectories with emergency-event onsets (vertical dotted lines marking the Accident, SignalLoss, and Overheated injection steps from the event log; BadWeather is routine and OperatorFocus operator-directed, so neither is marked). The channels show accumulate-on-trigger/decay-between dynamics across three distinct channels; H_c_ is dormant under routine patrol and recruited only in Sc3 (the sustained-distress episodes that recruit H_c_ occur only under multi-stressor load (Sc3). (**B**) α_eff_ tracks the stress channel inversely while remaining inside the personality envelope [0.456, 0.684]. H_c_ is shown as a logged readiness signal and is not behaviorally coupled to α_eff_.

**Figure 4:**
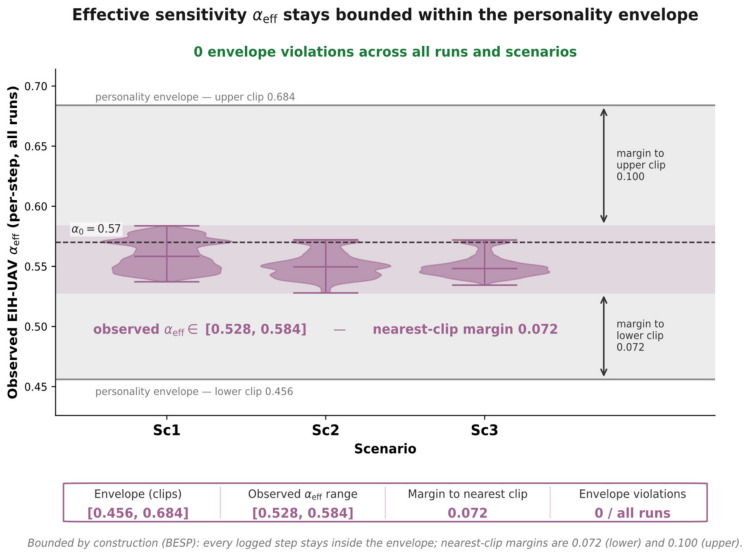
Bounded α_eff_ under the Personality-Preserving Bound (PPB). Allowed envelope [α_0_ (1 − ρ), α_0_ (1 + ρ)] = [0.456, 0.684] in light gray; observed EIH-UAV per-step α_eff_ distribution (all single-agent main-study runs, by scenario) as violins; α_0_ = 0.57 reference. Observed range [0.528, 0.584] lies entirely inside the envelope; nearest-clip margins are 0.072 (lower) and 0.100 (upper), with zero envelope violations across all runs and scenarios.

**Figure 5:**
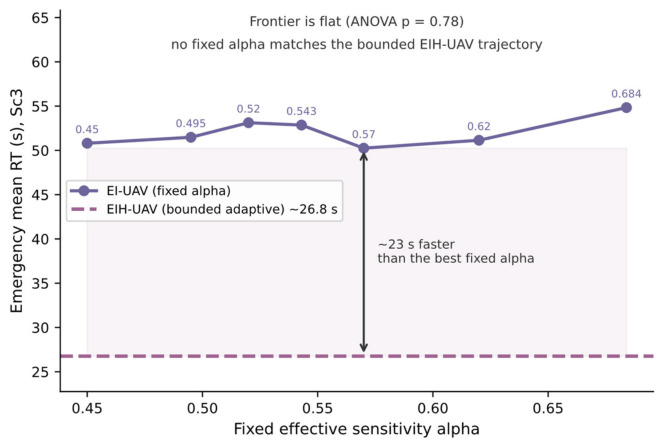
Bounded adaptation versus fixed sensitivity (Dataset C, Sc3). Emergency means RT across fixed effective-sensitivity values; the frontier is flat (one-way ANOVA *p* = 0.78), and the bounded EIH-UAV controller (26.8 s) sits every fixed value below, indicating the advantage arises from the bounded adaptive trajectory rather than any single sensitivity magnitude.

The within-run lag-1 autocorrelation of H_s_ is high (r ≈ 0.99), so the effective sample size is far below the raw per-step count, and the per-step correlations are treated as descriptive context, not inferential evidence. The per-run α_eff_ variance is small (SD = 0.0065 to 0.0135), reflecting tight controller convergence under bounded adaptation. Figure 3 shows α_eff_ and H_s_ trajectories over a representative Sc3 run, illustrating the asymmetric accumulation-decay dynamic and the bounded behavior guaranteed by BESP. Figure 4 visualizes the bounded behavior of α_eff_: the observed range [0.528, 0.584] across all single-agent main-study runs lies entirely inside the personality envelope [0.456, 0.684], with substantial margin to both clip bounds. EI-Low-UAV operates at fixed α = 0.495 by construction—below the EIH-UAV Sc3 operating band [0.534, 0.572], so the envelope characterization rests on the EIH-UAV trajectory data from the main study. A fixed-α frontier across the envelope (Figure 5) shows the emergency-response advantage is flat across fixed sensitivities (one-way ANOVA *p* = 0.78, η^2^ = 0.02, ω^2^ ≈ 0) and that the bounded EIH-UAV trajectory outperforms every fixed value.

**Table 8:** α_eff_ dynamics. EIH-UAV per-run mean ± SD; α_0_ = 0.57 fixed for EI-UAV.

Scenario	EI α	EIH α_eff_ (Mean ± SD)	Per-Step Range (All Runs Pooled)	Δα from α_0_	r(H_s_, α_eff_), n ≈ 13,400
Sc1	0.5700	0.5597 ± 0.0013	[0.537–0.584]	−1.81%	−0.790
Sc2	0.5700	0.5506 ± 0.0026	[0.528–0.572]	−3.40%	−0.598
Sc3	0.5700	0.5500 ± 0.0032	[0.534–0.572]	−3.51%	−0.447

The mechanism chain is sustained operational-load exposure, dominated by baseline traffic congestion and reinforced by event stressors, accumulates H_s_ (Sc1-Sc3 means 0.195–0.279) → α_eff_ settles at time-averaged 0.553 → reduced sensitivity raises the activation threshold for marginal signals → sustained focus at high-priority zones and faster arrival when emergencies occur. Relative to the uncoupled baseline, increasing the coupling coefficient reduced inter-agent sensitivity distance (from 0.00502 at γ = 0 to 0.00243 at γ = 0.50; one-way ANOVA F = 82.5, *p* < 0.0001; a 51.6% reduction), with both agents remaining within the personality envelope at every level (no violations).

**Disentangling warmup vs. steady-state contribution.** Across all Sc3 emergency detections (instant responses excluded), the median detection step is ≈1503. At detection, mean H_s_ is 0.318, but the stored α_eff_ sits at 0.554—only 2.9% below α_0_ and still 0.059 above EI-Low’s fixed 0.495 (the earliest emergencies, accidents detected mid-warmup near step 479, sit at 0.568, essentially α_0_); the 100-step update interval with λ = 0.10 (Section 3.2.5) holds α_eff_ near α_0_ at the moment of detection. Therefore, the adaptive controller detects emergencies at a higher sensitivity than the failing below-band control, so the advantage arises through the pre-detection trajectory (patrol history and zone occupancy under slow-ramping α_eff_) rather than from a numerical α gap at the moment of decision (Section 6.3).

### 5.5. Strategy Concentration and Hormone State

Strategy distributions across controllers under Sc3 are summarized in Table 9.

Hormone dynamics and PPE state distribution for the EIH-UAV controller are reported in Table 10.

Under multi-stressor load (Sc3), EIH-UAV commits to its dominant strategy more strongly than any other emotional controller: it spends 49.5% of steps in the S6 (Operator-Directed) strategy and shows the lowest switching entropy among the emotional controllers (1.78 bits, versus 1.85 for EI-UAV, 1.86 for EI-Low-UAV, and 2.05 for the ablated ABL-UAV). This focused commitment is attributable to the strategy-hold persistence channel: in the leave-one-out channel ablation (build-192 campaign, Section 6.1), removing the strategy-hold channel significantly lowers dominant-strategy occupancy (S6 47.0% to 44.8%; paired Wilcoxon *p* = 0.004), while the same removal leaves emergency response time statistically unchanged (Section 6.1). Therefore, the two stress-driven channels carry separable behavioral signatures: emergency amplification governs response latency, whereas strategy-hold governs behavioral persistence. RB-UAV operates near determinism (entropy 0.27 bits, S1 dominant 95.3% of steps), whereas the ablated ABL-UAV shows the highest switching entropy (2.05 bits), consistent with unbounded reactive switching once the personality envelope is removed. MHR-UAV concentrates comparably on S6 (49.2%, entropy 1.53 bits) under the coupled multi-agent regime. Six of the eight strategies (S0, S1, S2, S3, S4, S6) were active across all evaluated runs, consistent with the reachable strategy set under the Calm personality profile.

H_s_ remains moderately accumulated across all scenarios (means of 0.195, 0.279, and 0.270 for Sc1, Sc2, Sc3, respectively), producing the consistent α_eff_ reduction in Table 8 through k_s_ = ρ · α_0_. The reward channel (H_r_), which encodes coverage success, is active near-continuously (H_r_ > 0.001 in 98.7–100% of steps across scenarios) at a modest time-averaged level (means 0.08–0.11), reflecting the steady accrual of coverage successes under the Calm profile rather than sparse pulses. The reward channel is wired into the sensitivity update and is empirically success-responsive: following emergency resolution, Hr rose in all 24 Sc3 runs (mean ΔHr = +0.048, 95% CI [+0.043, +0.053], Wilcoxon *p* = 1.2 × 10^−7^), consistent with its success-triggered design. The operator-engagement channel (H_c_) is dormant under routine patrol and is recruited only in Sc3 (mean 0.016, with zero activation in Sc1–Sc2; active in 29% of Sc3 steps), where sustained distress (negative mood), not the operator-focus event itself, recruits the engagement response, which rises and decays within bounds without saturating. The present scenarios include no reward-amplification structures that would elevate H_r_ further; full evaluation of the reward pathway requires scenarios with such structures (Section 7).

Operator-engagement channel (Hc): functionality and selectivity. The operator-engagement hormone Hc is recruited selectively under sustained distress. It is strictly silent in Sc1 and Sc2 (0 of 24 runs) and recruited only under multi-stressor load in Sc3 (19 of 24 runs; mean 0.016, peak 0.189, active in 29% of steps). Recruitment is driven by negative mood rather than by the operator-focus event itself: the per-step rate of change in Hc tracks falling pleasure, with a run-level correlation r (ΔHc, pleasure) = −0.75 (mean across runs, 95% CI [−0.80, −0.69]; all 19 recruited runs negative, range −0.85 to −0.50; Wilcoxon *p* = 3.8 × 10^−6^). This driver is specific to the engagement channel: the stress channel Hs is essentially flat against pleasure (−0.05) and tracks arousal instead, while the two hormone levels are only weakly correlated (+0.17). Consistent with its design, Hc remains below the operator-status action threshold throughout (peak 0.189 versus a 0.50 mode-switch threshold), the rapport mode never changes (Independent in 100% of steps), and Hc’s behavioral output is unwired in single-agent operation. We therefore make no single-agent performance claim for Hc; the analysis establishes that the channel is functional and selectively gated rather than decorative (Table 11). The per-run statistics (Appendix D) are reported descriptively and are consistent with the distress-integrating update specified for the channel, not as independent mechanistic validation.

### 5.6. Activation-Selectivity Control

To rule out the alternative explanation that EIH-UAV’s Sc3 advantage reflects a fixed lower α-value rather than the dynamic trajectory through which EIH arrives at that value, the EI-Low-UAV control was run: an EI-UAV variant with α fixed at 0.495 throughout the mission—below the EIH-UAV Sc3 operating band [0.534, 0.572]—holding all other architecture, scenario definitions, seeds, and event schedules identical to the main study. EI-Low therefore tests the steady-state-α hypothesis with a value more favorable to it than EIH’s own: a fixed sensitivity even lower than the band EIH occupies, but with no dynamic trajectory.

The EI-Low control does not reproduce EIH’s response-time advantage. In Sc3, EI-Low-UAV achieves 51.1 ± 10.2 s, not significantly different from EI-UAV at α = 0.57 (49.2 ± 9.6 s; Mann–Whitney U = 252, *p* = 0.47 two-sided; |r_pb| = 0.12). EIH-UAV is 1.91× faster than EI-Low (Section 5.1), a result insensitive to instant-response handling (1.93× when the instant-response runs are treated as 0 s). Behavior in Sc3 is α-trajectory-dependent, not α-value-dependent. Strategy distributions corroborate fixed-α parity (Table 9: EI-Low-UAV S6 occupancy 42.7% vs. EI-UAV 44.8%; entropy 1.86 vs. 1.85 bits). The alternative interpretation that the DHL collapses to a “good fixed α” is therefore not supported: no fixed α at the EIH grand-mean reproduces the EIH advantage in this evaluation (Figure 5).

All conclusion-bearing comparisons reported in this section are collected, with their test statistic, *p*-value, effect size, and 95% confidence interval, in Table 12.

## 6. Discussion

### 6.1. Mechanism: From Hormone to Behavior

The present study provides two complementary forms of evidence for the DHL: mechanism-level evidence at the per-step scale through the H_s_-to-α_eff_ relationship (Section 5.4), and behavioral-level evidence through response-time separation under sustained multi-stressor exposure (Section 5.1). The behavioral result (Section 5.1) is the basis for distinguishing the Sc3 separation from sampling noise; the per-step relationship (Section 5.4) is consistent with the implemented coupling and is reported as a mechanistic context, not as independent evidence.

The H_s_ → α_eff_ → behavior chain is inspired by a biological regulatory motif: cortisol-associated regulation under sustained operational demand can attenuate reactivity to marginal stimuli, allowing organisms to focus resources on high-priority demands. The DHL replicates this computationally. The biological analogies motivate the accumulation-decay structure and the timescale separation between fast affective dynamics and slow hormonal modulation; they are not intended as a claim of physiological fidelity. Across all evaluated scenarios, congestion exposure and event-driven stress drive H_s_ to a sustained mean near 0.25, which through k_s_ = ρ · α_0_ produces a time-averaged α_eff_ of 0.553 (a 2.9% reduction from α_0_ = 0.57; the observed 0.553 mean exceeds the nominal 0.542 by the early-mission warmup ramp documented in Section 5.4). The result is a sustained suppression of the environmental-appraisal weight in Equation (1), dampening oscillatory zone transitions and lengthening dwell at high-priority locations. The bounded magnitude of H_s_ (means 0.195–0.279 across Sc1–Sc3) reflects the λ_s_ << δ_s_ asymmetry chosen in Section 3.2: fast accumulation under congestion exposure drives H_s_ rapidly to its sustained, de-saturated plateau (well below the envelope ceiling) during the early-mission ramp, while slow decay (λ_s_ = 0.005) holds it at that bounded level across the rest of the mission, regardless of how many discrete events the scenario injects.

**Why Sc3 specifically?** The Sc3 multi-event load provides two ingredients absent from Sc1 and Sc2: (i) sustained dwell at high-priority zones produced by reduced α_eff_, and (ii) multiple injection opportunities for arrivals to land adjacent to a current dwell zone. Together, these convert the trajectory-shaped patrol history into a measurable adjacency-translation advantage between events. Sc1 lacks events; Sc2 has a single event with no second arrival to benefit from adjacency; only Sc3 provides both ingredients. Therefore, the additional Sc3 stressors act less as primary H_s_ generators (H_s_ rises only modestly with scenario load and stays within its bounded de-saturated band, from 0.195 in Sc1 to 0.270 in Sc3; Table 8) and more as temporal opportunities through which the trajectory-shaped patrol bias becomes behaviorally observable. The mechanism is active in all scenarios, but its statistical signature requires Sc3’s structure to emerge. The EI-Low-UAV control argues against steady-state-α as a sufficient explanation (Section 5.6), and Section 5.4 per-step relationship is consistent with the implemented coupling (reported descriptively, not as an independent inferential test).

**Non-monotonicity of α and response time.** The relationship between α and response time is non-monotonic. EI-UAV (α = 0.57): 49.2 s; EI-Low-UAV (α = 0.495): 51.1 s; ABL-UAV (α = 1.0): 49.8 s; EIH-UAV (adaptive α_eff_, grand-mean 0.553): 26.8 s. The simple narrative “lower α produces longer dwell and faster response” predicts EI-Low—fixed at α = 0.495, below the EIH-UAV operating band [0.534, 0.572] and therefore the more favorable static value for that narrative—should at least approach EIH’s 26.8 s, yet it achieves only 51.1 s, not significantly different from EI-UAV at α = 0.57. A static sensitivity below the adaptive controller’s own operating range does not reproduce the advantage. The observed ordering is EIH ≪ ABL ≈ EI ≈ EI-Low. The DHL contribution is therefore not simply lower α: it combines pre-event trajectory-shaped dwell-bias with moment-of-detection emergency amplification (Equation (5)), neither of which is available to fixed-α controllers.

### 6.2. Bounded Adaptation as a Safety Property

The PPB ρ = 0.20 enforces a mathematical guarantee that effective sensitivity remains within a predefined behavioral envelope at every step. The observed α_eff_ range across all EIH-UAV single-agent main-study runs is [0.528, 0.584], well inside [0.456, 0.684]. The bounding is achieved by coefficient construction (k_s_ = k_r_ = ρ·α_0_ = 0.114) rather than clip activation; the clip in Equation (3) is a second-line guarantee never activated in nominal operation. The construction supports envelope-based verification under the evaluated mission histories and scenario conditions.

### 6.3. Activation Selectivity as a Designed Property

EIH-UAV’s emergency-response advantage appears wherever emergency events occur: it is 1.54× faster than EI-UAV in Sc2 (*p* < 0.001) and 1.84× faster in Sc3 (*p* < 0.001), with no significant difference in the ratio between scenarios, while routine events are not accelerated (Section 5.1); the selectivity is event-type, not scenario-level. Although α_eff_ modulation is present in all scenarios (Table 8), the behavioral acceleration is concentrated on emergency events. The deployment consequence: a controller that adapts indiscriminately would amplify noise and destabilize routine operation, while one whose behavioral change is concentrated on emergency events and gated by accumulated operational context is more resistant to routine over-adaptation. The DHL achieves this passively through the slow accumulation timescale of H_s_, without a separate scenario detector or mode switch.

### 6.4. Robustness of the Bounded Effective Sensitivity Property Under Inter-Agent Coupling

The MHR-UAV (Multi-agent Hormone-Regulated UAV) extension adds inter-agent stress coupling (Equation (8)), introducing only one additional scalar parameter γ. The principal claim of the multi-agent extension is robustness of the Bounded Effective Sensitivity Property (BESP) under inter-agent coupling: across coupling levels, the personality envelope (Equation (3) bounds) remains binding for both agents independently, so coupling does not push either agent outside the envelope. This is an envelope-preservation claim, not a claim of performance improvement over the single-agent controller. Asymmetric configurations and three-or-more-agent extensions are deferred to future work (Section 7).

Adding a second coupled agent preserves the Bounded Effective Sensitivity Property (BESP) while requiring only one additional scalar parameter. The MHR mean emergency response (49.8 s, instant-excluded) is substantially faster than the rule-based baselines; because the multi-agent and single-agent results are drawn from separate campaigns, no direct comparison with the single-agent controller is asserted. The Sc3 severe-zone coverage is consistent with both agents prioritizing similar high-severity zones under symmetric coupling (reduced spatial diversification), an explicit trade-off of symmetric coupling. The bounded-by-construction property propagates from the single-agent to the multi-agent configuration without parameter overhead.

**Figure 6:**
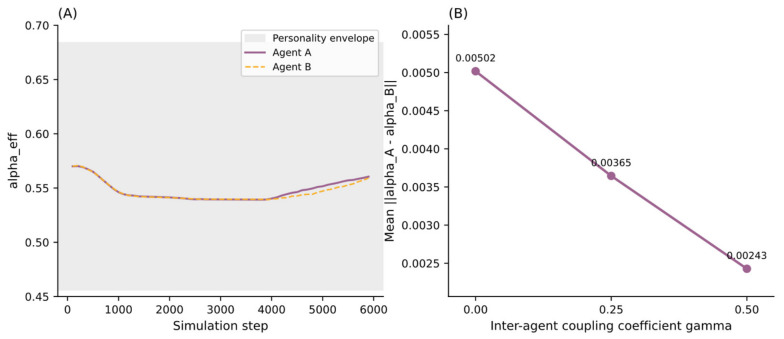
Multi-agent coupling under varied coupling strength for MHR-UAV (Sc3). (**A**) Per-step effective-sensitivity trajectories of Agent A and Agent B for one representative run, overlaid on the personality envelope [0.456, 0.684]. (**B**) Inter-agent sensitivity distance as a function of the coupling coefficient γ (0.00, 0.25, 0.50), with 95% confidence intervals; the distance decreases as coupling increases while both agents remain within the envelope.

To evaluate the effect of coupling strength, the coupling coefficient was varied across γ = 0.00 (uncoupled), 0.25, and 0.50 in the multi-stressor scenario (24 seeds per level). As summarized in Table 13, increasing γ reduced the inter-agent sensitivity distance from 0.00502 (γ = 0) to 0.00243 (γ = 0.50), a 51.6% reduction (one-way ANOVA F = 82.5, *p* < 0.0001), while both agents remained within the personality envelope at every level (Figure 6). The inter-agent sensitivity distance decreased monotonically as the coupling coefficient increased, and across all tested γ values, no envelope violations were observed.

**Table 13:** Multi-agent coupling statistics. MHR-UAV inter-agent sensitivity distance ‖α_A_ − α_B_‖ across coupling strength γ (Sc3, 24 seeds per level; step-aligned).

γ	n Runs	Mean ‖α_A_ − α_B_‖	95% CI	Envelope Violations
0.00 (uncoupled)	24	0.00502	[0.00462, 0.00541]	0 of 24
0.25	24	0.00365	[0.00338, 0.00391]	0 of 24
0.50	24	0.00243	[0.00225, 0.00261]	0 of 24

The envelope-preservation result has a concrete deployment consequence: the parameter-level envelope guarantee established for the single-agent EIH controller (BESP, Section 3.2.4) carries over to the MHR multi-agent configuration because the per-step coupling preserves the personality envelope across all tested coupling levels, with no envelope violations observed. This does not establish system-level multi-agent safety: deadlock potential, inter-agent interaction patterns, and the analogous full closed-loop multi-agent stability argument are not addressed by the present convergence result and require separate analysis (Section 7). The cost of adding a second coupled agent at γ ≤ 0.50 is therefore an additive scalar parameter that preserves the parameter-level envelope, not a re-derivation of system-level multi-agent guarantees. Asymmetric γ values and three-or-more-agent extensions remain to be evaluated (Section 7).

### 6.5. Design Properties: Mission-Final Battery and Coverage Trade-Off

The introduction of adaptive sensitivity reshapes the observed trade-off under the evaluated scenarios. Under multi-stressor load (Sc3), EIH-UAV delivers faster emergency response (1.91× over EI-Low, Section 5.1) with comparable mission-final battery consumption across controllers (Section 5.3); no battery-efficiency advantage is claimed. Total in-flight energy across single-agent controllers in Sc3 is comparable (138–140% gross, Table A3). The strategy distribution under multi-stressor load (Table 9) supports this interpretation: EIH-UAV concentrates on S6 (Operator Directed, 49.5%) and S2 (Weighted Priority, 30.2%), the most focused single-strategy commitment among the emotional controllers, suppressing the rapid local switches characteristic of the higher-entropy ABL-UAV. Therefore, DHL improves emergency response without a battery-efficiency penalty under the evaluated scenarios.

The remaining genuine trade-off concerns coverage breadth versus personality preservation. ABL-UAV (45.8% SZD in Sc3) reaches Severe-zone dwell at parity with EIH at the cost of operating without the personality-restoration term entirely (α = 1.0, no bounded envelope), trading bounded-by-construction behavioral identity for unbounded reactivity. EIH-UAV (45.7%) reaches Severe-zone dwell statistically at parity with ablated control (45.8%) while keeping α_eff_ within ±20% of α_0_. The 4 percentage-point gap relative to ABL is the cost of personality preservation (any α < 1.0) rather than of DHL adaptation specifically: fixed-α emotional control (EI-UAV, 41.6%) achieves comparable Severe-zone dwell to EIH (45.7%), so the DHL itself adds essentially no coverage cost over fixed-α emotional control.

MHR-UAV consumes 62.4% at Sc3, a separate-campaign readout reported without between-controller interpretation (the per-step drain is identical by construction). Taken together, with coverage retained within run-to-run variance and total in-flight energy similar across single-agent controllers, the DHL recovers most of the ablated controller’s coverage benefit and delivers faster response time with comparable mission-final battery consumption relative to fixed-α emotional control (Section 5.3). The bounded-by-construction safety property is preserved throughout.

## 7. Limitations and Future Work

**Scope of the bounded-adaptation guarantee.** The Bounded Effective Sensitivity Property establishes that the effective sensitivity remains within the personality envelope under every admissible hormone state. This is a parameter-level safety property, not a closed-loop stability proof for UAV motion or a mission-level safety certificate. Full closed-loop stability under affect-driven control, including the interaction between the effective-sensitivity dynamics and the host PAD-mood-strategy decision chain, requires Lyapunov-style analysis or formal verification of the integrated controller-environment system and is outside the scope of the present work. The DHL is therefore best interpreted as a bounded parameter-regulator with a designed safety envelope, not as a verified flight-safe controller.

**Statistical scope and sample size.** The load-bearing comparisons use 24 independent runs per controller-scenario cell. Per-run aggregates are analyzed with non-parametric tests and reported with effect sizes and bootstrap confidence intervals, and the family of pairwise comparisons is Holm-corrected. Statistics are computed at the run level rather than on pooled per-step samples, so within-run autocorrelation does not inflate the effective sample size of any reported inference. The aggregate findings should be interpreted as strong simulation evidence rather than definitive operational validation. Where per-step quantities are described, they are reported as descriptive characterizations of the controller dynamics, not as independent inferential evidence. A power calculation confirms the design is adequately powered for its headline claims: at 24 runs per cell, a two-sided test at α = 0.05 and 80% power detects standardized effects of |d| ≥ 0.81. The emergency-response advantages exceed this bound by a wide margin (Hedges g = −2.9 for EIH-UAV versus EI-Low-UAV and −2.8 for EIH-UAV versus EI-UAV in Sc3, and −1.2 in Sc2), so they are not power-limited. The EI-UAV versus EI-Low-UAV contrast has an observed effect of g = −0.18, well below the minimum detectable effect, so its non-significance reflects a genuinely small difference that the present sample cannot resolve, and it is reported as non-significant without asserting equivalence. Every comparison underlying a stated conclusion is reported with its test statistic, *p*-value, effect size (Hedges g for parametric contrasts or rank-biserial r for rank-based tests), and 95% bootstrap confidence interval, so practical significance can be judged independently of the *p*-value; comparisons that do not bear on a conclusion are reported descriptively. The full set of conclusion-bearing contrasts is consolidated in Table 12.

**Hormone parameters and the reward and engagement channels.** Hormone parameters and trigger thresholds were set by design rationale rather than learned or systematically optimized; this is an explicit limitation, and a learned or optimization-based parameterization is a direction for future work. Sensitivity sweeps over the smoothing coefficient, the update cadence, and the personality-preservation budget are reported and show that the main results are stable to these choices. The hormone decay sweep is reported separately as a regime characterization rather than a flat-response axis: the locked decay value places the controller in a de-saturated stress regime, while halving the decay rate re-enters hormone saturation by design, so the locked configuration is a deliberate bounded choice rather than a point of invariance across all decay values. A full joint optimization over the hormone-parameter space is not performed here. The reward and operator-engagement channels remain operationally subdominant under the evaluated scenarios; dedicated scenarios targeting reward and sustained operator-engagement conditions would be required to evaluate these channels more fully, and we do not make performance claims for them in the present study.

**Figure 7:**
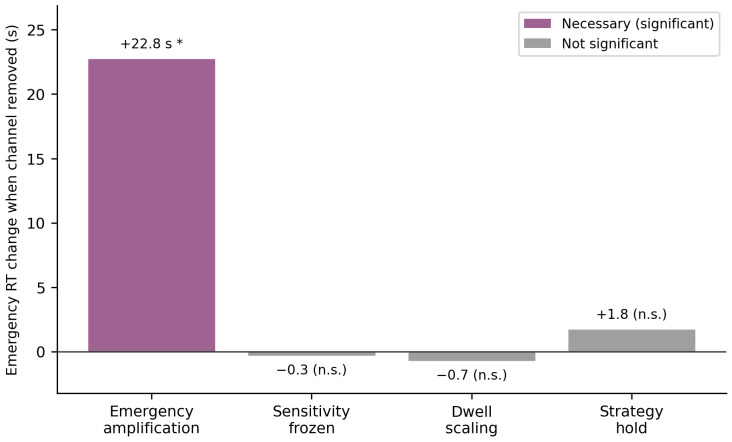
Channel necessity (leave-one-out ablation, Dataset B v192, Sc3). Change in emergency response time when each auxiliary channel is removed: only removing the emergency-event amplification channel degrades response (*: +22.8 s; Cohen d = 2.76; Holm-corrected *p* = 7.3 × 10^−5^); the dwell, sensitivity-freeze, and strategy-hold channels are not significant, establishing functional separability of the architecture.

**Channel attribution.** The relative contribution of the auxiliary H_s_-driven channels to the emergency response-time advantage is identified by a leave-one-out channel-ablation analysis (Section 5): removing the emergency-event amplification channel produces the largest and only statistically significant degradation after correction (Figure 7), while freezing sensitivity magnitude and removing the dwell-scaling and strategy-hold channels are each individually non-significant. This identifies the emergency-event amplification channel as the largest measurable contributor under the evaluated leave-one-out configuration, and characterizes sensitivity magnitude as a bounded regulator rather than the largest contributor to the advantage. Finer-grained decomposition within the emergency channel and interaction effects among channels remain directions for future work.

The present study evaluates RBplus-UAV as an event-aware reactive baseline rather than as a fully optimized rule-based controller. Systematic optimization of rule-based baselines (e.g., hyperparameter tuning of the event-detection thresholds and zone-priority weights) and characterization of such optimized baselines as a separate point of comparison remain future work.

**Multi-agent scope.** Multi-agent coupling is evaluated through a coupling-strength sweep against an uncoupled baseline, which establishes that the Bounded Effective Sensitivity Property (BESP) is preserved under symmetric coupling up to the evaluated coupling strength. This is a parameter-level robustness result, not a system-level multi-agent guarantee. Asymmetric coupling configurations, three-or-more-agent extensions, deadlock and oscillatory-interaction analysis, communication latency, and the full closed-loop multi-agent stability argument are not addressed here and require separate study before stronger multi-agent operational claims can be made.

**Generalization across networks, personalities, and mission profiles.** The evaluation uses a single urban network (Bologna), a single fixed event schedule, a single Calm personality profile, and a single mission duration. The DHL coefficients rescale with the personality baseline, so the absolute envelope width differs across personality profiles; per-profile evaluation is required to confirm generalization. Evaluation on additional networks (for example, suburban-grid and downtown-corridor topologies) and on additional testbeds, including hardware platforms such as the GRASP Multiple Micro-UAV Testbed, would strengthen external validity; the parameter-level, bounded-by-construction nature of the regulator may facilitate portability but does not establish it. The single 6000-step mission duration is also a limitation: behavior under substantially longer or shorter missions, and under repeated recharge cycles, is not characterized here and is left for future work.

**Battery and energy.** Mission-final and total in-flight battery consumption are comparable across the single-agent controllers under the evaluated scenarios; no battery-efficiency advantage is claimed. The energy model is therefore neutral with respect to the controllers within this study, and the battery figures are reported for completeness rather than as a controller benefit. Explicit modeling of the relationship between sustained engagement, coverage breadth, and end-of-mission charge reserves, including travel-distance accounting and battery-aware hormone modulation, is a direction for future work toward longer-duration mission profiles where charge reserves are critical.

Pre-registration of the EI-Low-UAV control. The post hoc origin of the EI-Low control is fully disclosed in Section 4.2 and Section 5.6. A pre-registered replication on an independent seed set, with the EI-Low α-value fixed before any EIH simulations are run and with explicit family-wise correction (e.g., Holm–Bonferroni) applied to the exploratory pairwise contrasts reported in Section 5.1, would strengthen the present targeted test into a formally controlled comparison. Broader α sweeps (e.g., a fixed-α grid over the full personality envelope [0.456, 0.684]) would further characterize the dynamics-versus-steady-state distinction across the entire DHL operating range.

**Robustness to noise, communication failure, and adversarial disturbance.** The evaluated scenarios do not include sensor noise, communication dropouts or failures, or adversarial disturbances. Robustness of the controller to these conditions is therefore not established by the present study and is a dedicated direction for future work; the bounded-by-construction nature of the regulator constrains the sensitivity response but does not by itself guarantee robustness to corrupted sensing or hostile inputs.

**Deployment and sim-to-real.** The evaluation is simulation-only. Real UAV deployment introduces communication latency, sensing uncertainty, wind disturbance, sensor noise, actuator delay, and onboard energy-model validation that the present simulation does not capture. The bounded-adaptation guarantee applies to the controller-level effective-sensitivity dynamics independently of the deployment medium, and the bounded-by-construction nature of the regulator may simplify a sim-to-real transition by preserving the same parameter envelope across simulation and physical deployment; however, the empirical magnitudes reported here, particularly the response-time results, require physical validation under real sensing and actuation uncertainty before extrapolation to operational deployment. The hormone update parameters and the update cadence were calibrated for a one-second simulation step; real control loops operating at higher rates would require parameter rescaling to preserve the same operational time constants, which appears straightforward analytically but should be validated rather than assumed. Established sim-to-real techniques, such as domain-randomized policy transfer to physical quadrotors [38], offer a concrete path toward this validation.

## 8. Conclusions

This paper introduced the Digital Hormone Layer (DHL), a neuroendocrine-inspired bounded regulator that makes a UAV’s emotional sensitivity context-aware during mission execution without modifying the established decision architecture. The proposed system establishes a three-timescale affect-driven controller, integrating personality (static, mission-invariant), emotion (per-step PAD dynamics), and hormones (slow context accumulation), with a PPB ρ = 0.20 that mathematically guarantees bounded effective-sensitivity modulation within a predefined personality envelope.

Three properties define the contribution. Architecturally, the three-timescale design provides a principled separation of concerns absent in existing affect-driven UAV control frameworks, integrating personality (static), emotion (per-step), and hormone (slow context accumulation) timescales within a single regulator. Mechanistically, the hormone-to-sensitivity chain is empirically demonstrated by a −2.9% effective-sensitivity reduction under multi-stressor conditions and a consistently inverse per-step Pearson correlation between accumulated stress and effective sensitivity (r = −0.79 to −0.45 across scenarios, reported as descriptive context given strong within-run autocorrelation; full statistical apparatus reported in Section 5.1 and Section 5.4). As a system property, activation selectivity is empirically demonstrated by an EI-Low-UAV control that fixes α at 0.495, below the EIH-UAV operating band, and does not reproduce the response-time advantage of the adaptive controller. EIH-UAV is 1.91× faster than EI-Low-UAV in Sc3 (95% bootstrap CI [1.70×, 2.14×]; full statistical apparatus reported in Section 5.1 and Section 5.6), while EI-Low and the standard EI baseline do not differ significantly (*p* = 0.47, rank-biserial r = −0.12, 95% CI [−0.44, 0.21]), supporting the conclusion that the DHL advantage derives from the dynamic trajectory of α_eff_ and the three auxiliary stress-driven channels disclosed in Section 3.2.6 rather than from a low steady-state α-value alone. The α-mediated trajectory (Equations (3) and (4)) constitutes the primary adaptive mechanism, operating on a per-step basis throughout the mission, with the emergency-event amplification channel carrying the dominant share of the effect (full decomposition in Section 6.1), while the dwell-scaling and strategy-hold channels are individually non-significant in ablation. Although α_eff_ modulation is mechanistically present across all scenarios, the DHL therefore selectively accelerates emergency response while preserving routine behavior, producing a significant response-time advantage under both single-incident (Sc2 1.54×) and sustained multi-stressor (Sc3 1.84×) conditions.

Beyond response-time improvement, mission-final and total in-flight battery consumption are comparable across the single-agent controllers, demonstrating that bounded sensitivity reduction improves response time without a battery-efficiency penalty under the evaluated scenarios. A single-equation multi-agent extension introduces only one additional scalar parameter γ and yields per-step α convergence well within the personality envelope; because the multi-agent and single-agent results are drawn from separate campaigns, no direct response-time comparison is asserted. System-level multi-agent properties (deadlock, inter-agent interaction effects, three-or-more-agent extensions) are not addressed by this convergence result and require separate analysis (Section 7). The strongest empirical claim of this study is therefore single-agent DHL activation selectivity under Sc3; multi-agent results are architectural feasibility evidence rather than fully validated system-level claims. The bounded adaptation guarantee positions DHL as a candidate architectural primitive for emotionally adaptive autonomous control, with broader operational assessment pending validation across networks, personality profiles, and coupling regimes (Section 7).

## Figures and Tables

**Table 1:** Positioning of the DHL against representative adaptive and bio-inspired control families. Columns correspond to the four design-space axes; the final column states the gap each family leaves that the DHL is constructed to fill.

Approach/Family	What Is Adapted	Timescale	Boundedness/Interpretability	Personality Preservation	Gap Relative to DHL
Bio-inspired airspace design (SMA corridors) [12]	Airspace structure (corridors) via swarm agents	Design-time	System-level, not agent-internal	N/A (no single-agent identity)	Adapts the airspace, not an agent’s internal sensitivity
Affective/PAD models in HRI [6,13,14,15,16]	Expressed emotional state	Variable	Affect as output, not a bound on behavior	None	Communicates affect; does not regulate or bound behavior
Adaptive gain scheduling [18,19]	Pre-tuned controller gains	Fast	Bounded but not affect-interpretable; no operational-history state in the gain-selection rule	None	Selects gains, not an interpretable affective parameter
MRAC/L1 adaptive control [20,21]	Controller parameters (error-nulling)	Fast	Stability-bounded; parameters not human-interpretable	None	Tracks a reference model rather than preserving an affective behavioral baseline
Meta-control [22]	Choice between controllers/strategies	Slow (arbitration)	Interpretable at the policy level only	None	Selects which policy, not how sensitive within bounds
Neuromodulated/emotion-modulated RL [3,23]	Learning hyperparameters, then learned policy	Learning-time	Typically learned and policy-centric	None	Bounded deployment-time sensitivity regulation is not explicit
PPE (fast affective regulator) [4]	Effective sensitivity (fast appraisal)	Fast	Bounded, interpretable	Personality baseline (fast loop)	Fast timescale only; no slow neuroendocrine modulation
Bio-inspired neuroendocrine controllers [29,30,31,32]	Action selection/power/expressive behavior	Slow	Partially interpretable	Not explicitly preserved	Isolated elements; no explicit personality envelope on an emotional architecture
Digital Hormone Layer (DHL), this work	Affective sensitivity (α_eff_), bounded within ±ρ	Slow (neuroendocrine)	Bounded-by-design; interpretable coefficients (k_s_, k_r_, k_c_, ρ)	Explicit personality-preservation envelope	(targets the previously unfilled region)

**Table 2:** Consolidated DHL parameters and inherited PPE baseline constants.

Parameter	Value	Description
α_0_	0.57	Baseline emotional sensitivity (Calm personality profile [4])
ρ	0.20	Personality Preservation Budget (envelope half-width)
β_0_	0.30	Baseline PAD attraction coefficient [4]
δ_s_/δ_r_/δ_c	0.10/0.10/0.03	Accumulation rates (Equation (2a–c))
λ_s_	0.005	Stress first-order decay rate (Equation (2a))
τ_r_/τ_c	100/400	Reward/operator-engagement decay constants (Equation (2b,c))
k_s_/k_r_	0.114/0.114	Stress/reward sensitivity coefficients (= ρ·α_0_)
k_c	0.30	Operator-channel amplification coefficient
k_emg	0.30	Emergency-response amplification (Equation (5))
k_high/k_low	0.80/0.60	Dwell scaling coefficients (Equation (6a,b))
k_T	1.0	Strategy-hold persistence scale (Equation (7))
γ	0.50	Multi-agent symmetric stress-coupling coefficient (Equation (8))

**Table 3:** Controller configurations.

Controller	Decision Logic	Sensitivity α	Hormone Layer
RB-UAV	Rule-based, fixed patrol routes	None	None
RBplus-UAV	Rule-based, event-triggered (S0/S1/S6)	None	None
EI-UAV	PAD + PPE, personality-initialized	Fixed (0.57)	None
EI-Low-UAV	PAD + PPE, activation-selectivity control	Fixed (0.495)	None
ABL-UAV	PAD + PPE with α = 1.0 (baseline-restoration ablated)	Fixed (1.0)	None
EIH-UAV	PAD + PPE + DHL, full hierarchy	Dynamic α_eff_	H_s_, H_r_, H_c_
MHR-UAV	DHL sensitivity-regulation core with inter-agent stress coupling (γ = 0.50)	Dynamic α_eff_	H_s_ coupled

**Table 4:** Scenario definitions.

Scenario	Description	Events	Injected Stressors
Sc1	Incident-free baseline traffic scenario	None	0
Sc2	Single accident with recovery	Accident (step 500, 500 steps)	1
Sc3	Multi-stressor: full hazard complement	Accident + SignalLoss + OperatorFocus + Overheated + BadWeather	5

**Table 5:** Emergency response time. Mean ± SD seconds, emergency events (3-type); Sc1 omitted. Per-cell n in Section 5.1. MHR-UAV reported separately in Section 6.4 (separate campaign; not directly comparable).

Scenario	RB-UAV	RBplus-UAV	EI-UAV	EI-Low-UAV	ABL-UAV	EIH-UAV	MHR-UAV
Sc2	199.3 ± 59.7	100.0 ± 20.5	44.0 ± 15.3	43.9 ± 16.0	38.6 ± 11.2	28.5 ± 9.1	—
Sc3	113.2 ± 27.7	63.2 ± 12.1	49.2 ± 9.6	51.1 ± 10.2	49.8 ± 8.3	26.8 ± 5.8	—

**Table 6:** Severe-zone dwell percentage (SZD%). Per-run mean ± SD. Computed per Equation (9). MHR is evaluated under Sc3 only (separate campaign); per-agent Severe-zone dwell 21.5% load; coverage is distributed, not duplicated. See Section 5.2.

Scenario	RB-UAV	RBplus-UAV	EI-UAV	EI-Low-UAV	ABL-UAV	EIH-UAV	MHR-UAV
Sc1	7.3 ± 0.5	15.2 ± 1.1	47.7 ± 6.1	48.3 ± 9.8	45.2 ± 11.1	45.2 ± 8.7	—
Sc2	13.6 ± 14.7	23.8 ± 18.3	56.5 ± 12.3	56.3 ± 15.4	52.9 ± 12.2	52.3 ± 10.5	—
Sc3	10.8 ± 4.3	18.7 ± 4.0	41.6 ± 6.9	42.4 ± 8.5	45.8 ± 7.4	45.7 ± 8.6	21.5 ± 9.2

**Table 9:** Strategy distribution. Per-controller, Sc3.

Strategy (% of Steps)	RB-UAV	RBplus-UAV	EI-UAV	EI-Low-UAV	ABL-UAV	EIH-UAV	MHR-UAV
S0	4.7	4.9	7.0	7.0	7.0	6.9	1.6
S1	95.3	82.6	15.5	15.6	13.5	10.2	14.0
S2	0.0	0.0	30.6	32.5	46.5	30.2	35.1
S3	0.0	0.0	2.2	2.2	13.6	3.2	0.0
S4	0.0	0.0	0.0	0.0	0.3	0.0	0.0
S6	0.0	12.5	44.8	42.7	19.1	49.5	49.2

**Table 10:** DHL hormone dynamics and PPE state distribution. EIH-UAV only.

Metric	Sc1	Sc2	Sc3
H_s_ mean	0.195	0.279	0.270
H_r_ mean	0.105	0.082	0.097
H_c_ mean	0.000	0.000	0.016
H_r_ active steps (% > 0.001)	99.9%	98.7%	100.0%
PPE REGULATING %	93.2%	78.2%	57.8%
PPE RECOVERING %	6.6%	12.7%	23.6%
PPE OVERRIDE %	0.2%	9.1%	18.6%

**Table 11:** Operator-engagement channel (Hc) recruitment summary (EIH-UAV, Sc3, 24 runs).

Property	Hc Result
Sc1/Sc2 activation	0/24 runs (silent)
Sc3 activation	19/24 runs
Mean/peak (Sc3)	0.016/0.189
Active steps (Sc3)	29%
Run-level r(ΔHc, pleasure)	−0.75 [−0.80, −0.69]; 19/19 runs negative; *p* = 3.8 × 10^−6^
Hs–Hc level correlation	+0.17
Operator-mode flip	0% (peak 0.189 < 0.50 threshold)

**Table 12:** Statistical contrasts underlying the stated conclusions (run-level, n = 24). Test statistic, *p*-value (Holm-corrected within each family; † marks the four-channel leave-one-out ablation family), effect size, and 95% bootstrap confidence interval. RT = emergency response time (3-type emergency set, instant-excluded); SZD% = severe-zone dwell percentage (Equation (9)); S6 occupancy = dominant-strategy occupancy; r = rank-biserial correlation; g = Hedges g; d = Cohen d; Δ = mean difference versus the reference condition.

Contrast (Metric, Scenario)	Test	Statistic	*p*	Effect Size	95% CI
EIH vs. EI-Low—RT, Sc3	Mann–Whitney	U = 10	1.05 × 10^−8^	g = −2.88; ratio 1.91×	[−4.03,−2.21]; [1.70,2.14]
EIH vs. EI—RT, Sc3	Mann–Whitney	U = 10	1.04 × 10^−8^	g = −2.78	[−3.77,−2.17]
EIH vs. EI—RT, Sc2	Mann–Whitney	U = 127	9.2 × 10^−4^	g = −1.21	[−1.95,−0.65]
EIH vs. EI-Low—RT, Sc2	Mann–Whitney	U = 123	6.8 × 10^−4^	g = −1.16	[−1.77,−0.68]
EI vs. EI-Low—RT, Sc3	Mann–Whitney	U = 252	0.47	r = −0.12	[−0.44, 0.21]
EIH vs. EI—SZD%, Sc3	Mann–Whitney	U = 378	0.063	r = 0.31	[−0.01, 0.61]
EI-Low vs. EI—SZD%, Sc3	Mann–Whitney	U = 302	0.78	r = 0.05	[−0.30, 0.38]
NOEMG vs. NONE—RT, Sc3	paired Wilcoxon	W = 0	7.3 × 10^−5^ †	d = 2.76; Δ +22.8 s	[+19.2, +26.2] s
ALPHAFROZEN vs. NONE—RT, Sc3	paired Wilcoxon	W = 15	0.67 †	d = −0.05; Δ −0.3 s	[−2.3, +1.6] s
NOHOLD vs. NONE—S6 occupancy, Sc3	paired Wilcoxon	W = 42	0.004 †	r = 0.69; Δ +2.2 pp	[0.33, 0.93]
α-magnitude—RT, Sc3	one-way ANOVA	F = 0.54	0.78	η^2^ = 0.02; ω^2^ ≈ 0	—
EIH vs. best static α (0.57)—RT, Sc3	Mann–Whitney	—	<0.001	g = −2.54; gap 23.5 s	[−3.74,−1.82]
ρ-magnitude—RT, pooled Sc2 + Sc3	one-way ANOVA	F = 0.03	0.97	η^2^ ≈ 0; ω^2^ ≈ 0	—

**Figure A1:**
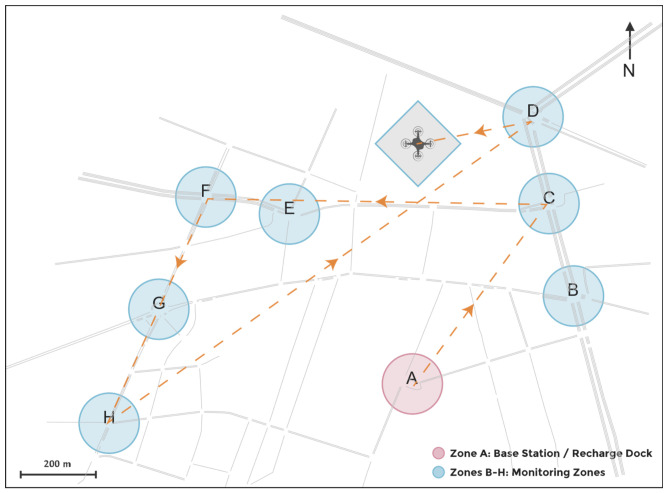
Bologna Acosta arterial network with eight monitoring zones (A–H, 80 m radius). Zone A is co-located with the base station and recharge dock; zones B–H are monitoring zones placed at principal arterial intersections. The UAV icon marks an in-flight patrol position; the dashed orange path is an illustrative non-sequential patrol trajectory (not derived from a specific simulation run).

**Figure A2:**
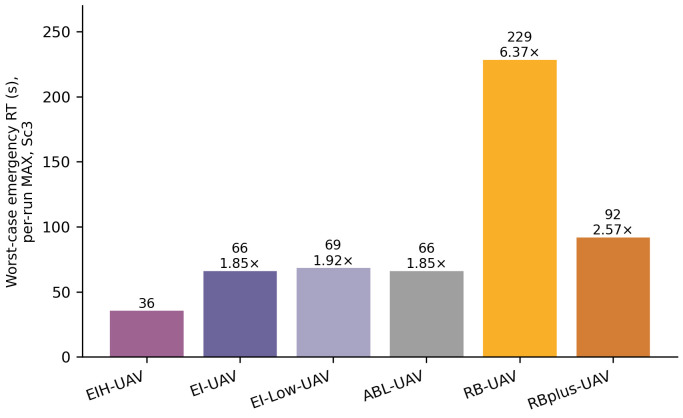
Per-run maximum emergency response time (Sc3, 3-type emergency, instant-excluded). EIH-UAV is 1.85× tighter than EI-UAV (Hedges g = −4.18); RB-UAV reaches 228.7 s.

**Figure A3:**
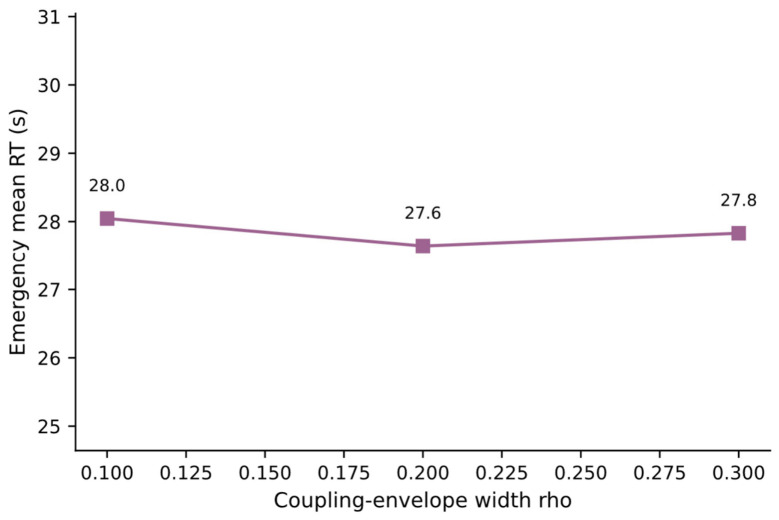
Emergency mean RT across coupling-envelope widths ρ (Dataset D, pooled Sc2 + Sc3). Emergency response is invariant to ρ (one-way ANOVA *p* = 0.97).

## Data Availability

The data presented in this study are available on request from the corresponding author.

## Abbreviations

The following abbreviations are used in this manuscript:

ABL	Ablated Baseline (Controller)
BESP	Bounded Effective Sensitivity Property
DHL	Digital Hormone Layer
EI	Emotionally Intelligent (UAV)
EI-Low	Emotionally Intelligent with Low Fixed α (Control)
EIH	Emotionally Intelligent Hormone-regulated (UAV)
EMA	Exponential Moving Average
HPA	Hypothalamic–Pituitary–Adrenal (axis)
MHR	Multi-agent Hormone-Regulated (UAV)
OCC	Ortony–Clore–Collins (appraisal model)
OCEAN	Openness–Conscientiousness–Extraversion–Agreeableness–Neuroticism
PAD	Pleasure–Arousal–Dominance
PPB	Personality Preservation Budget
PPE	Pull–Push Engine
RB	Rule-Based (controller)
RBplus	Rule-Based plus (event-aware controller)
Sc1	Scenario 1 (routine traffic monitoring)
Sc2	Scenario 2 (single accident event)
Sc3	Scenario 3 (multi-stressor exposure)
SUMO	Simulation of Urban MObility
SZD	Severe-zone dwell (percentage)
UAV	Unmanned Aerial Vehicle

## Appendix A. Bologna Network and Scenario Specification

This appendix provides the simulation environment specification for reproducibility: the SUMO Bologna Acosta arterial network with eight monitoring zones (Figure A1, Table A1), congestion classification thresholds, and the Sc3 event injection schedule (Table A2). The simulator-of-record is the same Bologna scenario introduced in [4] and extended in the present study to a six-controller comparison.

**Table A1:** Zone coordinates and roles. All zones have an 80 m monitoring radius.

Zone	Center (x, y) m	Role
A	(1130, 337)	Base station/recharge dock
B	(1559, 580)	East monitoring zone
C	(1494, 836)	North monitoring zone
D	(1452, 1076)	Northeast monitoring zone
E	(803, 807)	West monitoring zone
F	(581, 854)	South monitoring zone (accident locus)
G	(456, 544)	Southeast monitoring zone
H	(324, 228)	Northwest monitoring zone

**Congestion classification.** Congestion classes are defined by vehicle count within each zone’s 80 m monitoring radius: Severe (≥8 vehicles), High (4–7), Moderate (2–3), and Low (0–1). Thresholds are calibrated for the Bologna network baseline traffic load. Each simulation step represents one second of simulated time; missions run for 6000 steps, and the recharge cycle is triggered at 20% battery with linear restoration at 0.2% per step (up to 500 steps to restore from 20% to 100%).

**Table A2:** Sc3 event injection specifications. Per-event jitter of plus/minus 100 steps is applied per seed.

Event	Step	Duration	Target zone
Accident	500	500	F (fixed)
SignalLoss	1500	150	UAV current zone
OperatorFocus	2000	200	C (fixed)
Overheated	2600	200	UAV current zone
BadWeather	3000	200	Global

## Appendix B. In-Flight Energy Summary

Table A3 reports the full in-flight energy decomposition for single-agent controllers under Sc3 (multi-stressor scenario). MHR-UAV is omitted from the in-flight rows as it operates in the multi-agent coupling regime; per-agent energy in MHR is reported separately in Table 7 (mission-final values).

**Table A3:** In-flight energy summary (Sc3, single-agent controllers; MHR-UAV omitted from in-flight rows since it operates in the multi-agent coupling regime).

Controller	RB	RBplus	EI	EI-Low	ABL	EIH
In-flight (%)	139.1	139.1	140.0	140.0	140.1	138.7

## Appendix C. Worst-Case Emergency Response

Under the worst single emergency event per run (3-type emergency set {Accident, SignalLoss, Overheated}, Sc3, instant responses excluded), EIH-UAV holds the per-run maximum emergency response time 1.85× tighter than EI-UAV (Hedges g = −4.18; paired Wilcoxon *p* < 0.001, n = 24). The RB-UAV baseline reaches 228.7 s on the same worst-case measure (Figure A2). This is the per-run maximum, not a 90th-percentile statistic, and uses the same 3-type emergency set as the main analysis.

Emergency response is invariant to the envelope half-width ρ: across ρ = 0.10/0.20/0.30 (Dataset D, pooled Sc2 + Sc3), emergency mean RT is flat (one-way ANOVA *p* = 0.97, η^2^ ≈ 0), confirming the result is not an artifact of the ρ = 0.20 setting (Figure A3).

## Appendix D

Table A4 reports the per-run operator-engagement (Hc) recruitment statistics for the EIH-UAV controller in the multi-stressor scenario (Sc3, 24 seeds). Hc is dormant (—) in 5 of 24 runs and recruited in the remaining 19; in every recruited run, the per-step rate of change in Hc is negatively correlated with pleasure (distress-driven accumulation), with run-level r ranging from −0.50 to −0.85. Per-run correlations are reported descriptively (within-run pooled); the inferential claim is the across-run sign consistency (19 of 19 negative; Wilcoxon *p* = 3.8 × 10^−6^).

**Table A4:** Per-run Hc recruitment and distress correlation (EIH-UAV, Sc3).

Run	Hc Peak	Active Steps (%)	R (ΔHc, Pleasure)
1	0.164	36.7	−0.77
2	0.184	37.6	−0.80
3	0.177	37.1	−0.81
4	0.182	37.3	−0.78
5	—	—	dormant
6	—	—	dormant
7	—	—	dormant
8	0.176	37.1	−0.81
9	0.184	37.7	−0.82
10	0.110	34.2	−0.50
11	—	—	dormant
12	0.189	37.7	−0.85
13	0.130	35.0	−0.59
14	0.153	36.1	−0.78
15	0.114	34.0	−0.50
16	0.174	37.0	−0.76
17	0.183	37.5	−0.80
18	—	—	dormant
19	0.158	36.2	−0.77
20	0.173	37.1	−0.80
21	0.184	37.4	−0.81
22	0.188	37.9	−0.83
23	0.133	35.2	−0.65
24	0.165	36.8	−0.74
